# Supplementary material for: Intrinsic randomness in epidemic modelling beyond statistical uncertainty
Source: Commun Phys. 2023 Jun 20;6(1):146. doi: 10.1038/s42005-023-01265-2 (PMC11041706; doi:10.1038/s42005-023-01265-2)
Supplement: Supplementary file 2 — Supplementary Information [file 42005_2023_1265_MOESM2_ESM.pdf]

Supplementary Material: Intrinsic Randomness in Epidemic Modelling Beyond Statistical Uncertainty

Matthew J. Penn[1,*], Daniel J. Laydon[2], Joseph Penn[1], Charles Whittaker[2], Christian Morgenstern[2], Oliver Ratmann[2], Swapnil Mishra[3], Mikko S. Pakkanen[4,2], Christl A. Donnelly[1], and Samir Bhatt[2,3,*]

[1]University of Oxford

[2]Imperial College London

[3]University of Copenhagen

[4]University of Waterloo

[*]corresponding authors

Contents

1 Background literature on renewal equations **4**

2 Modelling **5**

2.1 Branching process framework 5

2.2 The counting process, $N(t, l)$ 6

2.3 The rate function, $r(t, l)$ 7

2.4 Smoothness assumptions . . . 7

2.5 Special cases for $N(t, l)$ . . . 8

3 Probability generating functions 8

3.1 General case . . . 8

3.2 Solving the pgf equation . . . 11

3.3 Poisson case . . . 11

3.4 Inhomogeneous Negative Binomial case . . . 13

3.5 Cumulative incidence . . . 15

3.6 A simplified pgf ignoring g . . . 15

3.7 Calculating the probability mass function via the pgf . . . 16

4 Properties of the prevalence variance 17

4.1 Derivation of equation for mean prevalence . . . 17

4.2 Derivation of equation for prevalence variance . . . 19

4.3 An explanation of the variance equation . . . 21

4.4 Overdispersion . . . 23

4.5 Comparison to a Poisson case . . . 29

4.6 Large time solutions to the variance equation . . . 30

4.7 Mean and variance for cumulative incidence . . . 33

5 Likelihood functions 33

5.1 Continuous case 33

5.2 Special case (Poisson) 36

5.3 Special case (Negative Binomial) 36

5.4 Approximating the likelihood 37

6 Assessing future variance during an epidemic 38

6.1 Derivation 38

6.2 Bounding the equation 44

6.3 Special cases 45

7 Discrete epidemics 46

7.1 Discrete pgf 46

7.2 Recovery of the continuous case 47

7.3 Distinctness from the continuous case 49

7.4 Discrete likelihood 50

Summary

This supplement provides full derivations of the results from the main text. The results are, as in the main text, presented for an epidemic occurring in continuous time, although some additional results on discrete epidemics are given in the final note of this supplement. The supplement is structured as follows.

- The first note, “Modelling”, provides a precise definition of the branching process model used

throughout the paper.

- The second note, "Probability generating functions" derives probability generating functions (pgfs) for prevalence and cumulative incidence. It also discusses their efficient solution, including some special cases in which one can speed up the solution process
- The third note, "Properties of the prevalence variance", derives the equation for the variance (via the previously derived equations for the pgf) and explores its properties, providing explanations for the various terms and proving that the prevalence of new infections is (under a mild condition on the possible spread of the epidemic) overdispersed.
- The fourth note, "Likelihood functions" contains the derivations of the pgf of the infection event times and the likelihood function presented in the main text.
- The fifth note, "Assessing future variance during an epidemic" derives the equation for variance of future cases when the cumulative incidence is known at some point in time.
- Finally, the sixth note, "Discrete epidemics" provides a range of similar results in the discrete setting, and shows the convergence of the pgf to its continuous equivalent as the step-size tends to zero.

Supplementary Note: 1 Background literature on renewal equations

A common approach to modelling infectious diseases is to use the renewal equation. The early theory on the properties of the renewal equation can be found here [8]. Epidemiologically derived descriptions can be found here [5, 9] where the renewal equation is framed in an epidemiological framework with reference to infection processes. The link between the renewal equation and the popular susceptible-infected-recovered models can be found here [4]. The basics of branching processes can be found here [10]. In what follows, we will arrive at a renewal equation from first principles by first starting with the probability generating function of a general branching process.

Supplementary Note: 2 Modelling

2.1 Branching process framework

We present a general time-varying age-dependent branching process that is most similar to the general branching process initially proposed by Crump, Mode and Jagers [6, 7]. Following [14], in our process, we begin with a single individual infected at some time l whose infectious period is a random variable distributed by cumulative distribution function $G(\cdot, l)$, admitting a probability density $g(\cdot, l)$. During this individual's life length, the individual gives rise to an integer-valued random number of secondary infections according to a counting processes $\{N(t,l)\}_{t\geq l}$ ($\{N(t,l)\}$ is the number of secondary infections) where t is a global "calendar" time. The amount of time for which the individual has been infected before time t is therefore $t - l$.

For each infection event time - that is, for each v such that

$$v \in \left\{ u \leq t : \lim_{s\to u_-} (N(s,l)) \neq \lim_{s\to u_+} (N(s,l)) \right\} \tag{S.1}$$

we then define a random variable

$$Y(v,l) := \lim_{s\to v_+} (N(s,l)) - \lim_{s\to v_-} (N(s,l)) \tag{S.2}$$

to be the size of the infection event at time v; that is, this is the number of individuals that are infected (by the initial individual) at time v. Throughout this paper, it will be assumed that $Y = Y(v)$, so that Y does not depend on the length of time for which an individual has been infected. However, this assumption could be removed from the model if desired.

Each newly infected individual then proceeds, independently, in the same way as the initial individual. The only change is that the time at which they are infected will be different (but, for example, the infection tree rooted at an individual infected at time $s > l$ is equal in distribution to the full infection tree if one started an epidemic with $l = s$). This self-similarity property underpins the derivations in the subsequent notes, as it allows an epidemic to be characterised purely by the "first generation" of infected individuals (and hence, the equations are derived using the "first generation principle").

2.2 The counting process, $N(t,l)$

Our framework relies on the assumption that the counting processes $N(t,l)$ has independent increments and is continuous in probability:

$$\lim_{\delta \to 0} \left[\mathbb{P}\Big(N(t+\delta, l) - N(t,l) \Big) \right] = 0 \quad \forall t \geq l \geq 0 \tag{S.3}$$

This condition excludes any discrete formulations of the epidemic process. It will be shown later in the supplement that discrete epidemics (which are not continuous in probability), are structurally different as extra terms appear in the equations for the pgf. However, the equations in the continuous case are recovered as the step-size of the discrete process tends to zero.

A further assumption on $N(t,l)$ is that it can be constructed from a Lévy Process - that is, there is some non-negative rate function $r(t,l)$ and some Lévy Process $\mathcal{N}(t)$ such that

$$N(t,l) = \mathcal{N}\Big(\int_l^t r(s,l) ds \Big) \tag{S.4}$$

Note that the counting processes relating to different individuals are independent, and hence will come from different independent copies of the base process $\mathcal{N}$.

This assumption is important because it means that the counting process of "infection events" (that is, points in time such that the value of $N(t,l)$ changes) is an inhomogeneous Poisson Process, which can be shown as follows. Consider a counting process, $J_{\mathcal{N}}(t,l)$ that counts the increases in $\mathcal{N}$. That is,

$$J_{\mathcal{N}}(t) := \left| \Big\{ u \leq t : \lim_{s \to u_-} (\mathcal{N}(s)) \neq \lim_{s \to u_+} (\mathcal{N}(s)) \Big\} \right| \tag{S.5}$$

where here $|\cdot|$ denotes the number of elements in a set. Then, as $\mathcal{N}$ is a Lévy Process, $J_{\mathcal{N}}(t)$ has iid (independent and identically distributed) increments and is non-decreasing in t with jumps of size 1 and thus follows a Poisson Process with some rate κ [1]. Thus, if $J(t,l)$ is the counting process of infection events in $\mathcal{N}(t,l)$, then

$$J(t,l) = J_{\mathcal{N}}\Big(\int_l^t r(s,l) ds \Big) \tag{S.6}$$

and hence, $J(t,l)$ is an inhomogeneous Poisson Process with rate $\kappa r(t,l)$ as required. In particular, defining

$$\lambda(t,l) := \int_l^t r(s,l)ds, \tag{S.7}$$

$J(t,l)$ has a generating function of

$$\mathcal{J}_{(t,l)}(s) = e^{\kappa\lambda(t,l)(s-1)} \tag{S.8}$$

2.3 The rate function, $r(t,l)$

Throughout the examples in this paper, the rate function $r(t,l)$ will be given as

$$r(t,l) = \rho(t)\nu(t-l) \tag{S.9}$$

Here, $\rho(t)$ is a population-level infection event rate. Note that, because the number of infections caused at each infection rate may be greater than 1 (that is one may have $J(t,l) < N(t,l)$), $\rho(t)$ cannot necessarily be interpreted in direct analogue to the reproduction number. $\nu(t-l)$ gives the infectiousness of an individual after it has been infected for time $(t-l)$. It will be assumed that $\int_0^\infty \nu(s)ds = 1$ so that it ρ can be interpreted as the infection event rate.

2.4 Smoothness assumptions

Note that, throughout the derivations of this paper, the smoothness of ρ, ν and g will not be explicitly considered when taking limits - it will be assumed that they are sufficiently smooth for "natural" results to hold. The authors believe that the results of this paper will hold for any piecewise continuous choices for these functions, although more detailed analysis would be needed to provide a rigorous proof of this. It is possible that they hold for much wider classes of functions, but this seems to the authors to be outside the realm of epidemiological interest, as it appears implausible that any of these functions would not be piecewise continuous in a realistic setting.

Moreover, it will be assumed that unique solutions to the equations for the pgf, mean and variance exist. Again, a proof of this property is beyond the scope of this work, although the classes of

equations presented in this paper are common across the literature, and it is likely that interested readers with a pure mathematical background could find applicable results to address this issue.

2.5 Special cases for $N(t,l)$

Throughout this paper, two special cases for $N(t,l)$ are considered - the case where $N(t,l)$ is itself an inhomogeneous Poisson Process, and the case where $N(t,l)$ is a Negative Binomial process. These were used to construct the figures in the paper and explanations as to how they can be used will be presented throughout this supplement.

Supplementary Note: 3 Probability generating functions

3.1 General case

Define $F(t,l;s) := E\left(s^{Z(t,l)}\right)$ to be the generating function of $Z(t,l)$. For simplicity of notation the dependence of F on s will be suppressed.

To derive the generating function $F(t,l)$, we condition on the infection period (lifetime) of the initial case, L.

$$E\left(s^{Z(t,l)}\right) = \int_0^\infty E\left(s^{Z(t,l)}\middle| L = u\right) g(u,l)du \tag{S.10}$$

$$= \int_{t-l}^\infty E\left(s^{Z(t,l)}\middle| L = u\right) g(u,l)du + \int_0^{t-l} E\left(s^{Z(t,l)}\middle| L = u\right) g(u,l)du \tag{S.11}$$

The counting process of the first individual, $N(t,l)$ is independent of this first individual's infection period L. If $L > t - l$ then this individual is still infectious and able to infect others at time t. Therefore, conditional on $L > t - l$, the number of people they have infected before time t is independent of L (as all infections from $N(s,l)_{l\leq s\leq t}$ are counted, irrespectively of the value of L). That is (the first term in Equation 11)

$$\int_{t-l}^\infty E\left(s^{Z(t,l)}\middle| L = u\right) g(u,l)du = \int_{t-l}^\infty E\left(s^{Z(t,l)}\middle| L \geq t - l\right) g(u,l)du \tag{S.12}$$

and hence, the first integral in Supplementary Equation S.11 can be simplified to give

$$E\left(s^{Z(t,l)}\right) = \left(1 - G(t-l,l)\right)E\left(s^{Z(t,l)}\middle|L \geq t-l\right) + \int_0^{t-l} E\left(s^{Z(t,l)}\middle|L = u\right)g(u,l)du \quad \text{(S.13)}$$

Let us consider the second part of Supplementary Equation S.11. Suppose first that $L = u$ for some $u < t - l$ so that the index case is no longer alive at time t. Thus, the number of infection events caused by the index case is given by $J(l + u, l)$.

Define the set of times at which these infected events occurred to be $\{K_1, ..., K_{J(l+u,l)}\}$ where here, importantly, the K_i are labelled in a random order (so it is not necessarily the case that $K_1 < ... < K_{J(l+u,l)}$). As J is an homogeneous Poisson Process and $N(t, l)$ is continuous in probability, the K_i are therefore iid with pdf (probability density function)

$$f_K(k) = \frac{r(l+k,l)}{\int_0^u r(l+s,l)ds} \quad \text{(S.14)}$$

It is perhaps helpful to note that this is the step which relies on N being continuous in probability. If this were not the case and $N(t, l)$ had non-zero probability of increasing at some time s, then the knowledge that $K_1 = s$ would give some information about K_2, as the fact that $K_2 \neq s$ would change its probability distribution, meaning K_1 and K_2 would not be independent. Conversely, in the continuous case, $K_1 = s$ removes an event of zero measure from the probability space of K_2, and hence K_1 and K_2 are still independent.

Now, by the self-similarity property ([10, 11]) we have

$$Z(t,l) = \sum_{i=1}^{J(l+u,l)} \sum_{j=1}^{Y(l+K_i(l+u,l))} Z_{ij}(t, l + K_i(l+u,l)) \quad \text{(S.15)}$$

where each Z_{ij} is an independent copy of Z that is equal in distribution. Z_{ij} denotes the jth individual corresponding to infection event time i. The two summations, from all previous infections, sum over all the infection events and their sizes. This summation is valid as each individual behaves independently once it has been infected.

Recall that if X_i are iid random variables (with a generating function, $G_X(s)$) and if Y is a non-negative integer-valued random variable (again with a generating function, $G_Y(s)$), then,

$$E\left(s^{\sum_{i=1}^{Y} X_i}\right) = G_Y(G_X(s)) \quad \text{(S.16)}$$

By defining $\mathcal{J}_{(t,l)}$ to be the generating function of $J(t,l)$, this relationship allows us to write $\mathbb{E}(s^{Z(t,l)}|L=u)$ as

$$\mathbb{E}(s^{Z(t,l)}|L=u) = \mathcal{J}_{(l+u,l)}\bigg(E\bigg[s^{\sum_{j=1}^{Y(l+K(l+u,l))} Z_j(t,l+K(l+u,l))}\bigg]\bigg) \tag{S.17}$$

where here, K is equal in distribution to the K_i. Conditioning on the value of K,

$$E\bigg[s^{\sum_{j=1}^{Y(l+K)} Z_j(t,l+K)}\bigg] = \int_0^u E\bigg[s^{\sum_{j=1}^{Y(l+k)} Z_j(t,l+k)}\bigg]\frac{r(l+k,l)}{\lambda(l+u,l)}dk \tag{S.18}$$

Thus, defining $\mathcal{Y}_{(l+k)}$ to be the generating function of $Y(l+k)$

$$E\bigg[s^{\sum_{j=1}^{Y(l+K)} Z_j(t,l+K)}\bigg] = \int_0^u \mathcal{Y}_{(l+k)}(F(t,l+k))\frac{r(l+k,l)}{\lambda(l+u,l)}dk \tag{S.19}$$

We can equivalently write this as an exponential, using the fact that $J(t,l)$ is Poisson distributed:

$$\mathbb{E}(s^{Z(t,l)}|L=u) = \mathcal{J}_{(l+u,l)}\bigg(\int_0^u \mathcal{Y}_{(l+k)}(F(t,l+k))\frac{r(l+k,l)}{\lambda(l+u,l)}dk\bigg) \tag{S.20}$$

$$= \exp\bigg[\kappa\lambda(l+u,l)\bigg(\int_0^u \mathcal{Y}_{(l+k)}(F(t,l+k))\frac{r(l+k,l)}{\lambda(l+u,l)}dk - 1\bigg)\bigg] \tag{S.21}$$

An identical derivation can be performed on the first integral in Supplementary Equation S.11 (swapping $t-l$ for u and multiplying by s to account for the initial case, which is counted in the prevalence at t when $L > t-l$), resulting in

$$\mathbb{E}(s^{Z(t,l)}|L \geq t-l) = s\mathcal{J}_{(t,l)}\bigg(\int_0^{t-l} \mathcal{Y}_{(l+k)}(F(t,l+k))\frac{r(l+k,l)}{\lambda(t,l)}dk\bigg) \tag{S.22}$$

$$= s\exp\bigg[\kappa\lambda(t,l)\bigg(\int_0^{t-l} \mathcal{Y}_{(l+k)}(F(t,l+k))\frac{r(l+k,l)}{\lambda(t,l)}dk - 1\bigg)\bigg] \tag{S.23}$$

and therefore, this yields an overall pgf

$$F(t,l) = s\Big(1-G(t-l,l)\Big)\mathcal{J}_{(t,l)}\bigg(\int_0^{t-l} \mathcal{Y}_{(l+u)}(F(t,l+u))\frac{r(l+u,l)}{\lambda(t,l)}du\bigg)...$$

$$... + \int_0^{t-l} \mathcal{J}_{(l+u,l)}\bigg(\int_0^u \mathcal{Y}_{(l+k)}(F(t,l+k))\frac{r(l+k,l)}{\lambda(l+u,l)}dk\bigg)g(u,l)du \tag{S.24}$$

or, equivalently

$$F(t,l) = s\Big(1-G(t-l,l)\Big)\exp\bigg[\kappa\lambda(t,l)\bigg(\int_0^{t-l} \mathcal{Y}_{(l+k)}(F(t,l+k))\frac{r(l+k,l)}{\lambda(t,l)}dk - 1\bigg)\bigg]...$$

$$... + \int_0^{t-l} \exp\bigg[\kappa\lambda(l+u,l)\bigg(\int_0^u \mathcal{Y}_{(l+k)}(F(t,l+k))\frac{r(l+k,l)}{\lambda(l+u,l)}dk - 1\bigg)\bigg]g(u,l)du \tag{S.25}$$

Note that by absorbing κ into the rate function $r(l+k,l)$, it can be assumed that $\kappa = 1$. Intuitively this is simply scaling the probability density by the number of points.

3.2 Solving the pgf equation

Practically, one will always set $l = 0$ for an epidemic, and so only the values $F(t, 0)$ are directly relevant. However, it is still necessary to solve for $F(t, l)$ for $0 \leq l \leq t$. In the language of PDEs (partial differential equations) and, specifically, the Cauchy problem, this can be explained by the fact that the "data curve" is the line $t = l$ (as the values of $F(t, t)$ are known to be equal to s) and the "characteristics" of the system are the lines $t =$ constant. Thus, to calculate the value of $F(t, 0)$, it is necessary to follow the characteristic from (t, t) to $(t, 0)$ and hence calculate $F(t, l)$ for $0 \leq l \leq t$.

Hence, following [14], solving Supplementary Equation S.25 can be greatly facilitated by defining an auxiliary equation $F_c(t) = F(c, c - t)$ and allows us to write Supplementary Equation S.25 an equation in one variable. This is

$$\begin{aligned} F_c(t) = s\Big(1 - G(t,l)\Big)\mathcal{J}_{(c,c-t)}\bigg(\int_0^t \mathcal{Y}_{(c-t+u)}(F_c(t-u))\frac{r(c-t+u,c-t)}{\lambda(c,c-t)}du\bigg)... \\ ... + \int_0^t \mathcal{J}_{(c-t+u,c-t)}\bigg(\int_0^u \mathcal{Y}_{(c-t+k)}(F_c(t-k))\frac{r(c-t+k,c-t)}{\lambda(u,c-t)}dk\bigg)g(u,l)du \end{aligned} \tag{S.26}$$

or, equivalently

$$\begin{aligned} F_c(t) = s\Big(1 - G(t,l)\Big)\exp\bigg[\lambda(c,c-t)\kappa\bigg(\int_0^t \mathcal{Y}_{(c-t+u)}(F_c(t-u))\frac{r(c-t+u,c-t)}{\lambda(c,c-t)}du - 1\bigg)\bigg]... \\ ... + \int_0^t \exp\bigg[\lambda(u,c-t)\kappa\bigg(\int_0^u \mathcal{Y}_{(c-t+k)}(F_c(t-k))\frac{r(c-t+k,c-t)}{\lambda(u,c-t)}dk - 1\bigg)\bigg]g(u,l)du \end{aligned} \tag{S.27}$$

3.3 Poisson case

If $N(t, l)$ is an inhomogeneous Poisson Process, then, as the infection event size for a Poisson Process is always 1 [1], one has $\mathcal{Y}_{(t)}(s) = s$. To aid understanding below in the Negative Binomial

case, it is helpful to note that the Lévy Process, $\mathcal{N}$, can hence be characterised by

$$\mathbb{P}(\mathcal{N}(t+dt)-\mathcal{N}(t)=0)=1-\kappa dt$$
$$\mathbb{P}(\mathcal{N}(t+dt)-\mathcal{N}(t)=1)=\kappa dt$$
$$\mathbb{P}(\mathcal{N}(t+dt)-\mathcal{N}(t)>1)=o(dt)$$

Setting $\kappa=1$ as discussed above, the generating function equation becomes

$$F(t,l)=s\Big(1-G(t-l,l)\Big)\exp\left[\Big(\int_0^{t-l}F(t,l+k)\rho(l+k)\nu(k)dk-\lambda(t,l)\Big)\right]... \tag{S.28}$$
$$...+\int_0^{t-l}\exp\left[\Big(\int_0^{u}F(t,l+k)\rho(l+k)\nu(k)dk-\lambda(l+u,l)\Big)\right]g(u,l)du \tag{S.29}$$

This equation can be further simplified by recalling that

$$\lambda(t,l):=\int_l^t r(u,l)du=\int_0^{t-l}r(u+l,l)du=\int_0^{t-l}\rho(u+l)\nu(u)du \tag{S.30}$$

therefore

$$F(t,l)=s\Big(1-G(t-l,l)\Big)\exp\left[\Big(\int_0^{t-l}F(t,l+k)\rho(l+k)\nu(k)dk-\int_0^{t-l}\rho(l+k)\nu(k)dk\Big)\right]...$$
$$...+\int_0^{t-l}\exp\left[\Big(\int_0^{u}F(t,l+k)\rho(l+k)\nu(k)dk-\int_0^{u}\rho(l+k)\nu(k)du\Big)\right]g(u,l)du$$

$$=s\Big(1-G(t-l,l)\Big)\exp\left[\Big(\int_0^{t-l}\rho(l+k)\nu(k)\big(F(t,l+k)-1\big)dk\Big)\right]...$$
$$...+\int_0^{t-l}\exp\left[\Big(\int_0^{u}\rho(l+k)\nu(k)dk\big(F(t,l+k)-1\big)\Big)\right]g(u,l)du \tag{S.31}$$

For computational ease the auxiliary function equation is then

$$F_c(t)=s\Big(1-G(t,l)\Big)\exp\left[\Big(\int_0^{t}\big(F_c(t-u)-1\big)\rho(c-t+u)\nu(u)du\Big)\right]...$$
$$...+\int_0^{t}\exp\left[\Big(\int_0^{u}\big(F_c(t-k)-1\big)\rho(c-t+k)\nu(k)dk\Big)\right]g(u,l)du \tag{S.32}$$

3.4 Inhomogeneous Negative Binomial case

Our derivation follows from the well-known relationship that the Negative Binomial distribution arises from a compound Poisson distribution. For $p \in (0,1)$ and $\phi \in \mathbb{R}^+$, if

$$X = \sum_{i=1}^{N} Y_i \tag{S.33}$$

where

$$N \sim \text{Poisson}(-\phi \ln(p)) \tag{S.34}$$

and each Y_i is independent of N, iid, and follows a logarithmic series distribution

$$Y_i \sim \text{Logarithmic}(1-p) \tag{S.35}$$

then the random variable X is Negative Binomial distributed. This can easily be proven using pgfs. Therefore we have $\kappa = -\ln(p)\phi$ and can calculate the pgf for Y as $\mathcal{Y}(s) = \frac{\ln(1-(1-p)s)}{\ln(p)}$. These can then be substituted into our general Supplementary Equation S.25.

For clarity we re-derive this relationship explicitly. We have

$$\mathcal{N}(t) \sim \text{NB}(\phi t, p) \tag{S.36}$$

As $M(t)$ has iid increments,

$$\mathbb{P}\Big(\mathcal{N}(t+dt)-\mathcal{N}(t) = k\Big) = \mathbb{P}\Big(\mathcal{N}(dt) = k\Big) = \frac{(k+\phi dt-1)(k+\phi dt-2)...\phi dt}{k!}(1-p)^k p^{\phi dt} \tag{S.37}$$

Thus, to leading order, for $k > 0$, one has

$$\mathbb{P}\Big(\mathcal{N}(t+dt)-\mathcal{N}(t) = k\Big) = \frac{(1-p)^k \phi dt}{k} + o(dt) \tag{S.38}$$

while if $k = 0$,

$$\mathbb{P}\Big(\mathcal{N}(t+dt)-\mathcal{N}(t) = 0\Big) = p^{\phi dt} = 1 + \ln(p)\phi dt + o(dt) \tag{S.39}$$

(noting that $\ln(p) < 0$). This means that the infection event process $J_{\mathcal{N}}$ satisfies

$$\mathbb{P}\Big(J_{\mathcal{N}}(t+dt) - J_{\mathcal{N}}(t) = 0\Big) = 1 + \ln(p)\phi dt + o(dt) \tag{S.40}$$

and

$$\mathbb{P}\Big(J_{\mathcal{N}}(t+dt)-J_{\mathcal{N}}(t)=1\Big)=\sum_{k=1}^{\infty}\frac{(1-p)^k\phi dt}{k}+o(dt) \tag{S.41}$$

$$=-\ln(p)\phi dt+o(dt) \tag{S.42}$$

and hence, $J_{\mathcal{N}}$ is a Poisson Process of rate $-\ln(p)\phi$ [2] . Thus, one has

$$\kappa=-\ln(p)\phi \tag{S.43}$$

as expected. Moreover, the pmf (probability mass function) of a infection event size, Y is given by

$$\mathbb{P}(Y=k)=\frac{(1-p)^k}{-k\ln(p)} \tag{S.44}$$

One can hence find the generating function as

$$\mathcal{Y}(s)=\sum_{k=1}^{\infty}\frac{((1-p)s)^k\phi}{-k\ln(p)} \tag{S.45}$$

Noting that

$$\sum_{k=1}^{\infty}\frac{(1-p)^k}{-k\ln(p)}=1 \tag{S.46}$$

one has

$$\mathcal{Y}(s)=\frac{\ln(1-(1-p)s)}{\ln(p)}\sum_{k=1}^{\infty}\frac{(1-(1-(1-p)s))^k}{-k\ln(1-(1-p)s)}=\frac{\ln(1-(1-p)s)}{\ln(p)} \tag{S.47}$$

These results can be substituted into the general formula to give

$$\begin{aligned}F(t,l)=&s\Big(1-G(t-l,l)\Big)\exp\Big[-\phi\Big(\int_0^{t-l}\ln(1-(1-p)F(t,l+u))\rho(u+l)\nu(u)du+\ln(p)\lambda(t,l)\Big)\Big]...\\&...+\int_0^{t-l}\exp\Big[-\phi\Big(\int_0^{u}\ln(1-(1-p)F(t,l+k))\rho(k+l)\nu(k)dk+\ln(p)\lambda(u,l)\Big)\Big]g(u,l)du\end{aligned} \tag{S.48}$$

As in the Poisson case, this equation can be simplified by factoring λ

$$\begin{aligned}F(t,l)=&s\Big(1-G(t-l,l)\Big)\exp\Big[-\phi\Big(\int_0^{t-l}\big(\ln(1-(1-p)F(t,l+u))-\ln(p)\big)\rho(u+l)\nu(u)du\Big)\Big]...\\&...+\int_0^{t-l}\exp\Big[-\phi\Big(\int_0^{u}\big(\ln(1-(1-p)F(t,l+k))-\ln(p)\big)\rho(k+l)\nu(k)dk\Big)\Big]g(u,l)du\end{aligned} \tag{S.49}$$

The easier-to-solve auxiliary function is given by

$$F_c(t) = s\Big(1 - G(t-l,l)\Big)\exp\Bigg[-\phi\Big(\int_0^t \big(\ln(1-(1-p)F_c(t-u)) - \ln(p)\big)\,\rho(c-t+u)\nu(u)du\Big)\Bigg]...$$
$$... + \int_0^t \exp\Bigg[-\phi\Big(\int_0^u \big(\ln(1-(1-p)F_c(t-k)) - \ln(p)\big)\,\rho(c-t+k)\nu(k)dk\Big)\Bigg]g(u,l)du \tag{S.50}$$

If $p = \frac{\phi}{1+\phi}$, then the Poisson case (with $\kappa = 1$) is recovered in the $\phi \to \infty$ limit.

Note that $\mathbb{E}[N(t,l)] = \frac{\phi\lambda(t,l)(1-p)}{p}$ while in our case, we impose that $\mathbb{E}[N(t,l)] = \lambda(t,l)$. Solving for p we can see $p = \frac{\phi}{1+\phi}$ and this relation can be substituted into Supplementary Equation S.50. Note that this agrees with the definition of p in the Poisson limit.

3.5 Cumulative incidence

Similar to prevalence, cumulative incidence can be calculated by counting all previous infections as well as current ones. Following an identical derivation to prevalence the pgf for cumulative incidence simply requires multiplying the second integral by s as the initial infection is counted in the cumulative incidence regardless of the value of L.

$$F(t,l) = s\Big(1 - G(t-l,l)\Big)\mathcal{J}_{(t,l)}\Big(\int_0^{t-l} \mathcal{Y}_{(l+u)}(F(t,l+u))\frac{r(l+u,l)}{\lambda(t,l)}du\Big)...$$
$$... + s\int_0^{t-l} \mathcal{J}_{(l+u,l)}\Big(\int_0^u \mathcal{Y}_{(l+k)}(F(t,l+k))\frac{r(l+k,l)}{\lambda(t,l)}dk\Big)g(u,l)du \tag{S.51}$$

3.6 A simplified pgf ignoring g

By assuming $g(u,l) = 0 \;\; \forall \;\; u$ and therefore $G(u,l) = 0 \;\; \forall \;\; u$, the pgf for prevalence (or, in this case, equivalently, cumulative incidence) simplifies to

$$F(t,l) = s\mathcal{J}_{(t,l)}\Big(\int_0^{t-l} \mathcal{Y}_{(l+u,l)}(F(t,l+u))\frac{r(l+u,l)}{\lambda(t,l)}du\Big)$$

Additional computational savings can be gained in our case $r(t,l) = \rho(t)\nu(t-l)$ if the infectiousness ν decays to zero quickly. This means that the auxiliary equation used for computation can be

truncated to some time $\min(t, T)$. For example, in the Poisson case this becomes,

$$F_c(t) = \exp\left[\left(\int_0^{\min(t,T)} \big(F_c(t-u) - 1\big)\, \rho(c - t + u)\nu(u)du\right)\right] s \tag{S.52}$$

and in the Negative Binomial case this becomes,

$$F_c(t) = \exp\left[-\phi\left(\int_0^{\min(t,T)} \big(\ln(1 - (1-p)F_c(t-u)) - \ln(p)\big)\, \rho(c - t + u)\nu(u)du\right)\right] s \tag{S.53}$$

These computational savings allow computation of the pgf for millions of iterations in minutes.

3.7 Calculating the probability mass function via the pgf

Following [13] and [3] (originally from [12]), by the properties of pgfs, the probability mass function p can be recovered through a pgf F's derivatives at $s = 0$

$$\mathbb{P}(n) = \frac{1}{n!}\left(\frac{d}{ds}\right)^n F(s; t, \tau)|_{s=0}$$

This is generally computationally intractable. A well-known result from complex analysis [12] holds that

$$f^{(n)}(a) = \frac{n!}{2\pi i} \oint \frac{f(z)}{(z-a)^{n+1}}\, dz. \tag{S.54}$$

Therefore

$$\mathbb{P}(n) = \frac{1}{2\pi i} \oint \frac{F(z; t, \tau)}{z^{n+1}} dz \tag{S.55}$$

This integral can be done on a closed circle around the origin such that $z = re^{i\theta}$ and $dz = izd\theta$ - i.e.

$$\mathbb{P}(n) = \frac{1}{2\pi} \int_0^{2\pi} \frac{F(re^{i\theta}; t, \tau)}{(re^{i\theta})^n} d\theta \tag{S.56}$$

Finally through substitution $\theta = 2\pi u$ such that $d\theta = 2\pi du$, where $u \in [0, 1]$ we find

$$\mathbb{P}(n) = \int_0^1 \frac{F(re^{2\pi iu}; t, \tau)}{r^n e^{2\pi iun}} du \tag{S.57}$$

Since trapezoidal sums are known to converge geometrically for periodic analytic functions (Davis 1959) a simple approximation becomes

$$\mathbb{P}(n) = \frac{1}{Mr^n} \sum_{m=0}^{M-1} F(re^{2\pi im/M}; t, \tau) e^{-2\pi inm/M} \tag{S.58}$$

Bornemann[3] suggest using $r = 1$.

The probability mass function for any time and n can be determined numerically. One needs $M \geq n$, which requires solving n renewal equations for the generating function and performing a fast Fourier transform. This is generally computationally fast, but may become slightly burdensome for epidemics with very large numbers of infected individuals.

Supplementary Note: 4 Properties of the prevalence variance

4.1 Derivation of equation for mean prevalence

Before deriving the equation for the prevalence variance, it is important to derive the equation governing the mean prevalence. This has been previously derived in [14], although here, we re-derive it from our new pgfs. First note that

$$\frac{\partial}{\partial s}\left(\mathcal{J}_{(t,l)}\left(\int_0^{t-l}\mathcal{Y}_{(l+u,l)}(F(t,l+u))\frac{r(l+u,l)}{\lambda(t,l)}du\right)\right)...$$
$$= \left[\int_0^{t-l}F_s(t,l+u)\frac{r(l+u,l)}{\lambda(t,l)}\mathcal{Y}'_{(l+u,l)}(F(t,l+u))du\right]\left[\mathcal{J}'_{(t,l)}\left(\int_0^{t-l}\mathcal{Y}_{(l+u,l)}(F(t,l+u))\frac{r(l+u,l)}{\lambda(t,l)}du\right)\right] \tag{S.59}$$

Now, setting $s = 1$ so that $F(\cdot,\cdot) = 1$ and $F_s(\cdot,\cdot) = M(\cdot,\cdot)$, one has

$$\left[\int_0^{t-l}M(t,l+u)\frac{r(l+u,l)}{\lambda(t,l)}\mathcal{Y}'_{(l+u,l)}(1)du\right]\left[\mathcal{J}'_{(t,l)}\left(\int_0^{t-l}\mathcal{Y}_{(l+u,l)}(1)\frac{r(l+u,l)}{\lambda(t,l)}du\right)\right] \tag{S.60}$$

Now, define $B(t) = \mathbb{E}(Y(t))$ so that $\mathcal{Y}'_{(l+u)}(1) = B(l+u)$. Moreover, $\mathcal{Y}_{(l+u)}(1) = 1$ so the equation becomes

$$\left[\int_0^{t-l}M(t,l+u)\frac{r(l+u,l)}{\lambda(t,l)}B(l+u)du\right]\left[\mathcal{J}'_{(t,l)}\left(\int_0^{t-l}\frac{r(l+u,l)}{\lambda(t,l)}du\right)\right] \tag{S.61}$$

Now, necessarily

$$\int_0^{t-l}\frac{r(l+u,l)}{\lambda(t,l)}du = 1 \Rightarrow \mathcal{J}'_{(t,l)}\left(\int_0^{t-l}\frac{r(l+u,l)}{\lambda(t,l)}du\right) = \mathbb{E}(J(t,l)) = \kappa\lambda(t,l) \tag{S.62}$$

and so, this results in

$$\int_0^{t-l} M(t,l+u)\frac{r(l+u,l)}{\lambda(t,l)}B(l+u)\kappa\lambda(t,l)du \tag{S.63}$$

Moreover, evaluating

$$\mathcal{J}_{(t,l)}\left(\int_0^{t-l}\mathcal{Y}_{(l+u)}(F(t,l+u))\frac{r(l+u,l)}{\lambda(t,l)}du\right) \tag{S.64}$$

at $s=1$ gives

$$\mathcal{J}_{(t,l)}\left(\int_0^{t-l}\mathcal{Y}_{(l+u)}(1)\frac{r(l+u,l)}{\lambda(t,l)}du\right) = \mathcal{J}_{(t,l)}\left(\int_0^{t-l} 1\times\frac{r(l+u,l)}{\lambda(t,l)}du\right) \tag{S.65}$$

$$= \mathcal{J}_{(t,l)}(1) \tag{S.66}$$

$$= 1 \tag{S.67}$$

Thus, the derivative of the full generating function equation gives

$$M(t,l) = (1-G(t-l,l))\left[1+\int_0^{t-l} M(t,l+u)\frac{r(l+u,l)}{\lambda(t,l)}B(l+u)\kappa\lambda(t,l)du\right]... \tag{S.68}$$

$$...+\int_0^{t-l}\int_0^u M(t,l+k)\frac{r(l+k,l)}{\lambda(l+u,l)}B(l+k)\kappa\lambda(l+u,l)g(u,l)dkdu \tag{S.69}$$

This can be simplified significantly. Note that,

$$\int_0^{t-l}\int_0^u M(t,l+k)\frac{r(l+k,l)}{\lambda(l+u,l)}B(l+k)\kappa\lambda(l+u,l)g(u,l)dkdu =$$
$$\int_0^{t-l}\int_0^u M(t,l+k)r(l+k,k)B(l+k)\kappa g(u,l)dkdu \tag{S.70}$$

Moreover, one can change the order of integration to get

$$\int_0^{t-l}\int_k^{t-l} M(t,l+k)r(l+k,k)B(l+k)\kappa g(u,l)dudk = \int_0^{t-l} M(t,l+k)r(l+k,k)B(l+k)\kappa(G(t-l,l)-G(k,l)) \tag{S.71}$$

and hence, one can write the equation for $M(t,l)$ as

$$M(t,l) = (1-G(t-l,l)) + \int_0^{t-l} M(t,l+u)r(l+u,l)\left(B(l+u)\kappa\right)(1-G(u,l))du \tag{S.72}$$

Note that, for the Poisson special case, $B(l+u,l)=1$ and for the Negative Binomial special case, $B(l+u,l)=\frac{p-1}{p\ln(p)}=-\frac{1}{\ln(p)\phi}$. In both cases, it may improve the epidemiological interpretation of ρ to absorb the $B(l+u,l)\kappa$ term into ρ (so that ρ becomes a measure of the rate of new infections). This gives the simpler equation

$$M(t,l) = (1-G(t-l,l)) + \int_0^{t-l} M(t,l+u)\rho(l+u)\nu(u)(1-G(u,l))du \tag{S.73}$$

which agrees with [14].

4.2 Derivation of equation for prevalence variance

The equation for variance can now be found by taking the second derivative of the pgf. Define $W(t,l) := \mathbb{E}(Z(t,l)(Z(t,l)-1))$. Note that this then gives the variance, $V(t,l)$ as $V(t,l) = W(t,l) + M(t,l) - M(t,l)^2$.

Consider first the term

$$s\Big(1 - G(t-l,l)\Big)\mathcal{J}_{(t,l)}\Big(\int_0^{t-l} \mathcal{Y}_{(l+u)}(F(t,l+u))\frac{r(l+u,l)}{\lambda(t,l)}du\Big) \tag{S.74}$$

The first derivative of this term is equal to

$$\begin{aligned}
&\bar{G}(t-l,l)\mathcal{J}_{(t,l)}\Big(\int_0^{t-l} \mathcal{Y}_{(l+u)}(F(t,l+u))\frac{r(l+u,l)}{\lambda(t,l)}du\Big) + ...\\
&s\bar{G}(t-l,l)\Big[\int_0^{t-l} F_s(t,l+u)\mathcal{Y}'_{(l+u)}(F(t,l+u))\frac{r(l+u,l)}{\lambda(t,l)}du\Big]\mathcal{J}'_{(t,l)}\Big(\int_0^{t-l} \mathcal{Y}_{(l+u)}(F(t,l+u))\frac{r(l+u,l)}{\lambda(t,l)}du\Big)
\end{aligned} \tag{S.75}$$

Then, the second derivative is equal to

$$\begin{aligned}
&2\bar{G}(t-l,l)\Big[\int_0^{t-l} F_s(t,l+u)\mathcal{Y}'_{(l+u)}(F(t,l+u))\frac{r(l+u,l)}{\lambda(t,l)}du\Big]\mathcal{J}'_{(t,l)}\Big(\int_0^{t-l} \mathcal{Y}_{(l+u)}(F(t,l+u))\frac{r(l+u,l)}{\lambda(t,l)}du\Big)+\\
&+ s\bar{G}(t-l,l)\Big[\int_0^{t-l} F_s(t,l+u)\mathcal{Y}'_{(l+u)}(F(t,l+u))\frac{r(l+u,l)}{\lambda(t,l)}du\Big]^2\mathcal{J}''_{(t,l)}\Big(\int_0^{t-l} \mathcal{Y}_{(l+u)}(F(t,l+u))\frac{r(l+u,l)}{\lambda(t,l)}du\Big)\\
&+ s\bar{G}(t-l,l)\Big[\int_0^{t-l} F_{ss}(t,l+u)\mathcal{Y}'_{(l+u)}(F(t,l+u))\frac{r(l+u,l)}{\lambda(t,l)}du\Big]\mathcal{J}'_{(t,l)}\Big(\int_0^{t-l} \mathcal{Y}_{(l+u)}(F(t,l+u))\frac{r(l+u,l)}{\lambda(t,l)}du\Big)\\
&+ s\bar{G}(t-l,l)\Big[\int_0^{t-l} F_s^2(t,l+u)\mathcal{Y}''_{(l+u)}(F(t,l+u))\frac{r(l+u,l)}{\lambda(t,l)}du\Big]\mathcal{J}'_{(t,l)}\Big(\int_0^{t-l} \mathcal{Y}_{(l+u)}(F(t,l+u))\frac{r(l+u,l)}{\lambda(t,l)}du\Big)
\end{aligned} \tag{S.76}$$

Now, one can evaluate this as $s = 1$. Note that

$$\begin{aligned}
\int_0^{t-l} \mathcal{Y}_{(l+u)}(F(t,l+u))\frac{r(l+u,l)}{\lambda(t,l)}du &= \int_0^{t-l} \mathcal{Y}_{(l+u)}(1)\frac{r(l+u,l)}{\lambda(t,l)}du\\
&= \int_0^{t-l} 1 \times \frac{r(l+u,l)}{\lambda(t,l)}du\\
&= 1
\end{aligned} \tag{S.77}$$

Moreover, define $B^W(t) := \mathbb{E}(Y(t)(Y(t)-1))$ and $C^W(t,l) := \mathbb{E}(J(t,l)(J(t,l)-1))$. Note also $\mathbb{E}(J(t,l)) = \lambda(t,l)$. Thus, the second derivative evaluated at $s=1$ is

$$\begin{aligned}
&2\bar{G}(t-l,l)\left[\int_0^{t-l} M(t,l+u)B(l+u)\frac{r(l+u,l)}{\lambda(t,l)}du\right]\kappa\lambda(t,l) \\
&+\bar{G}(t-l,l)\left[\int_0^{t-l} M(t,l+u)B(l+u)\frac{r(l+u,l)}{\lambda(t,l)}du\right]^2 C^W(t,l) \\
&+\bar{G}(t-l,l)\left[\int_0^{t-l} W(t,l+u)B(l+u)\frac{r(l+u,l)}{\lambda(t,l)}du\right]\kappa\lambda(t,l) \\
&+\bar{G}(t-l,l)\left[\int_0^{t-l} M(t,l+u)^2B^W(l+u)\frac{r(l+u,l)}{\lambda(t,l)}du\right]\kappa\lambda(t,l)
\end{aligned} \tag{S.78}$$

Noting that $J(t,l)$ is Poisson, one has

$$C^W(t,l) + \mathbb{E}(J(t,l)) - \mathbb{E}(J(t,l)^2) = \mathrm{var}(J(t,l)) = \mathbb{E}(J(t,l)) \tag{S.79}$$

and hence

$$C^W(t,l) = \mathbb{E}(J(t,l))^2 \tag{S.80}$$

Define

$$\chi(t,l,k) := \kappa\left[W(t,l+k)B(l+k)r(l+k,l) + M(t,l+k)^2B^W(l+k)r(l+k,l)\right] \tag{S.81}$$

Then, the same process can be carried out for the second part of the equation to give

$$\begin{aligned}
W(t,l) =& 2\bar{G}(t-l,l)\left[\int_0^{t-l} M(t,l+u)B(l+u)r(l+u,l)du\right] + \bar{G}(t-l,l)\int_0^{t-l}\chi(t,l,k)dk... \\
&... + \bar{G}(t-l,l)\left[\int_0^{t-l} M(t,l+u)B(l+u)\kappa r(l+u,l)du\right]^2 ... \\
&... + \int_0^{t-l}\left[\int_0^{u} M(t,l+u)B(l+u)\kappa r(l+u,l)du\right]^2 g(u,l)du... \\
&... + \int_0^{t-l}\int_0^{u}\chi(t,l,k)dkg(u,l)du
\end{aligned} \tag{S.82}$$

For ease of notation, define

$$S(t,l,u) := \left[\int_0^{u} M(t,l+k)B(l+k)\kappa r(l+k,l)dk\right]^2 \tag{S.83}$$

so that

$$\begin{aligned}
W(t,l) =& 2\bar{G}(t-l,l)\left[\int_0^{t-l} M(t,l+u)B(l+u)r(l+u,l)du\right] + \bar{G}(t-l,l)\int_0^{t-l}\chi(t,l,k)dk... \\
&... + \bar{G}(t-l,l)S(t,l,t-l) + \int_0^{t-l} S(t,l,u)g(u,l)du + \int_0^{t-l}\int_0^{u}\chi(t,l,k)dkg(u,l)du
\end{aligned} \tag{S.84}$$

Now, changing the order of integration in the final term (as was done in the derivation of the mean prevalence), this can be rewritten as

$$W(t,l) = 2\bar{G}(t-l,l)\Bigg[\int_0^{t-l} \kappa M(t,l+u)B(l+u)r(l+u,l)du\Bigg] + \bar{G}(t-l,l)S(t,l,t-l)...$$
$$... + \int_0^{t-l} S(t,l,u)g(u,l)du + \int_0^{t-l} \chi(t,l,k)\bar{G}(k,l)dk \tag{S.85}$$

From this, we can create an equation for $\mathbb{E}(Z(t,l)^2) := X(t,l) = W(t,l) + M(t,l)$ by defining

$$\chi^X(t,l,k) = \kappa\Bigg[X(t,l+k)B(l+k,l)r(l+k,l) + M(t,l+k)^2 B^W(l+k,l)r(l+k,l)\Bigg] \tag{S.86}$$

and then simply adding the equation for M to give

$$X(t,l) = \bar{G}(t-l,l) + 2\bar{G}(t-l,l)\Bigg[\int_0^{t-l} \kappa M(t,l+u)B(l+u)r(l+u,l)du\Bigg] + \bar{G}(t-l,l)S(t,l,t-l)...$$
$$... + \int_0^{t-l} S(t,l,u)g(u,l)du + \int_0^{t-l} \chi^X(t,l,k)\bar{G}(k,l)dk \tag{S.87}$$

Finally, to form the equation for the variance $V(t,l) = X(t,l) - M(t,l)^2$, note that

$$\chi^X(t,l,k) = \kappa\Bigg[X(t,l+k)B(l+k)r(l+k,l) + M(t,l+k)^2(\mathbb{E}(Y(l+k)^2) - B(l+k))r(l+k,l)\Bigg] \tag{S.88}$$

$$= \kappa\Bigg[V(t,l+k)B(l+k)r(l+k,l) + M(t,l+k)^2\mathbb{E}(Y(l+k)^2)r(l+k,l)\Bigg] \tag{S.89}$$

$$:= \chi^V(t,l,k) \tag{S.90}$$

and hence, subtracting $M(t,l)^2$ from both sides of the equation for $X(t,l)$ gives

$$V(t,l) = \bar{G}(t-l,l) + 2\bar{G}(t-l,l)\Bigg[\int_0^{t-l} \kappa M(t,l+u)B(l+u)r(l+u,l)du\Bigg] + \bar{G}(t-l,l)S(t,l,t-l)...$$
$$... + \int_0^{t-l} S(t,l,u)g(u,l)du + \int_0^{t-l} \chi^V(t,l,k)\bar{G}(k,l)dk - M(t,l)^2 \tag{S.91}$$

4.3 An explanation of the variance equation

There are two main sources of uncertainty in the infection process - the infectious period of an individual, and the number and timing of infections that occur during this infectious period. One

can show that the variance splits into three terms - one for each of these two sources of uncertainty from the initial individual, and one which propagates the uncertainty through the descendants of the initial individual.

Each term will be derived by assuming that all other parts of the model are deterministic. To begin, suppose that the infectious period of the initial individual is random but all other parts of the model are deterministic, so that, given that the initial individual is infectious at time $l+u$, it will infect $B(l+u)r(l+u,l)dt$ people in the interval $[u, u+dt]$ (note that this is an abstraction to illustrate the source of this variance, as it is impossible for non-integer numbers of infections to occur). Moreover, it is assumed that each of these individuals have given rise to exactly $M(t, l+u)$ infections at time t. Then, note that

$$\begin{aligned}
\mathrm{var}(Z(t,l)) &= \mathbb{E}(Z(t,l)^2) - \mathbb{E}(Z(t,l))^2 && \text{(S.92)}\\
&= \int_0^\infty \mathbb{E}(Z(t,l)^2|L=u)g(u,l)du - M(t,l)^2 && \text{(S.93)}\\
&= \int_0^{t-l}\left[\int_0^u M(t,l+k)B(l+k)r(l+k,l)dk\right]^2 g(u,l)du... && \text{(S.94)}\\
&... + \bar{G}(t-l,l)\left(1+\int_0^{t-l} M(t,l+k)B(l+k)r(l+k,l)dk\right)^2 - M(t,l)^2\\
&= \bar{G}(t-l,l) + 2\bar{G}(t-l,l)\int_0^{t-l} M(t,l+k)B(l+k)r(l+k,l)dk + ... && \text{(S.95)}\\
&... + \bar{G}(t-l,l)S(t,l,t-l) + \int_0^{t-l} S(t,l,u)g(u,l)du - M(t,l)^2
\end{aligned}$$

which recovers all the terms of the variance equation except for $\int_0^{t-l}\chi^V(t,l,k)\bar{G}(k,l)dk$.

Now, suppose that the infectious period of the initial individual is deterministic in the sense that they infect others at a rate of $r(l+k,l)\bar{G}(k,l)$, i.e. the expected rate at time $l+k$. Thus, the number of infection events in the interval $[l+(k-1)dt, l+kdt]$ is (to leading order in dt) a Poisson variable, A_k, with mean $r(l+k,l)\bar{G}(k,l)dt$ and hence the number of infections is that Poisson variable multiplied by $Y(l+k,l)$. Finally, note that, as before, any individuals born at

time $l+k$ will be assumed to deterministically cause $M(t,l+k)$ active infections at time t. Thus,

$$\begin{aligned}\mathrm{var}(Z(t,l)) &= \int_{k=0}^{k=t-l} \mathrm{var}(M(t,l+k)Y(l+k)A_k) && \text{(S.96)}\\ &= \int_{0}^{t-l} \mathbb{E}((M(t,l+k)Y(l+k)A_k)^2) - \int_{k=0}^{k=t-l} \mathbb{E}((M(t,l+k)Y(l+k)A_k))^2 && \text{(S.97)}\\ &= \int_{k=0}^{k=t-l} M(t,l+k)^2\mathbb{E}(Y(l+k)^2)\mathbb{E}(A_k^2) - \int_{k=0}^{k=t-l} B(l+k)^2 r(l+k,l)^2 \bar{G}(k,l)^2 dt^2 M(t,l+k)^2 && \text{(S.98)}\end{aligned}$$

Ignoring the dt^2 term as it has zero measure, and noting that Y and A_k are independent

$$\mathrm{var}(Z(t,l)) = \int_0^{t-l} M(t,l+k)^2\mathbb{E}(Y(l+k,l)^2)r(l+k,l)\bar{G}(k,l)dt \tag{S.99}$$

which is again a term from the variance equation.

The final term, $\int_0^{t-l} V(t,l+k)B(l+k)\bar{G}(k,l)r(l+k,l)dk$ denotes the propagation of uncertainty through future generations. Indeed, if the infection process of the initial individual (and its infectious period) are assumed to be fully deterministic, then one simply has

$$\mathrm{var}(Z(t,l)) = \int_0^{t-l} \mathrm{var}(Z(t,l+k))\mathbb{E}(\text{number of individuals born at } l+k) \tag{S.100}$$

which can easily be seen to give the correct term.

4.4 Overdispersion

For the purposes of this note, it is helpful to create the following definition

Expanded: An epidemic is called "expanded" at time t, if there is a non-zero probability that the prevalence, not counting the initial individual or its secondary infections, is non-zero.

In this note, it will be shown that, if $\tilde{Z}(t,l)$ is the prevalence of *new* infections (that is, the prevalence without counting the initial case) then if the epidemic is expanded at time t, $\tilde{Z}(t,l)$ is strictly overdispersed. That is

$$\mathrm{var}(\tilde{Z}(t,l)) > \mathbb{E}(\tilde{Z}(t,l)) \quad \text{or} \quad \mathbb{E}(\tilde{Z}(t,l+k))\rho(l+k,l)\nu(k)\bar{G}(k,l) = 0 \quad \forall k \in (0,t-l) \tag{S.101}$$

The second condition ensures that, at each k, either the likelihood of a new infection being caused at time $l+k$, or the probability of an individual who was infected at time $l+k$ causing subsequent infections whose infection tree has non-zero prevalence at time t, is zero. Hence, it is equivalent to the epidemic not being expanded at time t.

It is crucial to use $\tilde{Z}(t,l)$ rather than $Z(t,l)$, as otherwise the deterministic initial case means that, for early times, the prevalence is underdispersed (as, for example $\mathbb{E}(Z(l,l)) = 1$ and $\text{var}(Z(l,l)) = 0$). Moreover, the condition on the tertiary infections is necessary as, otherwise, if $N(t,l)$ is Poissonian, then $\tilde{Z}(t,l)$ is also Poissonian (and therefore not strictly overdispersed).

It is helpful to derive equations for the quantities for the mean $\tilde{M}(t,l)$ and the variance $\tilde{V}(t,l)$ of the new infection prevalence. This can be done by following the methods of the previous note. The derivations are mostly identical, and so will not be covered in detail. However, the key point is to note that the equation for the pgf, $\tilde{F}$, becomes

$$\tilde{F}(t,l) = \Big(1 - G(t-l,l)\Big)\mathcal{J}_{(t,l)}\bigg(\int_0^{t-l} \mathcal{Y}_{(l+u)}(\tilde{F}(t,l+u))\frac{r(l+u,l)}{\lambda(t,l)}du\bigg)...$$
$$... + \int_0^{t-l} \mathcal{J}_{(l+u,l)}\bigg(\int_0^{u} \mathcal{Y}_{(l+k)}(\tilde{F}(t,l+k))\frac{r(l+k,l)}{\lambda(l+u,l)}dk\bigg)g(u,l)du \tag{S.102}$$

as the factor of s in the first term is discarded. This equation can then be differentiated as before to show that

$$\tilde{M}(t,l) = (1 - G(t-l,l))\bigg[\int_0^{t-l} \tilde{M}(t,l+u)\frac{r(l+u,l)}{\lambda(t,l)}B(l+u)\kappa\lambda(t,l)du\bigg]...$$
$$... + \int_0^{t-l}\int_0^{u} \tilde{M}(t,l+k)\frac{r(l+k,l)}{\lambda(l+u,l)}B(l+k)\kappa\lambda(l+u,l)g(u,l)dkdu \tag{S.103}$$

and then rearranged to

$$\tilde{M}(t,l) = \int_0^{t-l} \tilde{M}(t,l+u)r(l+u,l)B(l+u)\kappa(1 - G(u,l))du \tag{S.104}$$

Defining $\tilde{S}$ as the analogue to S, by

$$\tilde{S}(t,l,u) = \bigg[\int_0^{u} \tilde{M}(t,l+k)B(l+k)\kappa r(l+k,l)dk\bigg]^2 \tag{S.105}$$

and using $\bar{G} = 1 - G$, the first of these equations can be written more succinctly as

$$\tilde{M}(t,l) = \bar{G}(t-l,l)\tilde{S}(t,l,t-l)^{0.5} + \int_0^{t-l} \tilde{S}(t,l,u)^{0.5}g(u,l)du \tag{S.106}$$

The equation for $\tilde{V}(t,l)$ can be calculated in a similar way. The only changes to the derivation are that the first term in Supplementary Equation S.78 is discarded to account for the discarded s in the pgf, and that when adding the mean to move from W to X (in analogue to Supplementary Equation S.87), one no longer needs to add the $\bar{G}(t-l,l)$ term. Thus,

$$\tilde{V}(t,l) = \bar{G}(t-l,l)\tilde{S}(t,l,t-l) + \int_0^{t-l} \tilde{S}(t,l,u)g(u,l)du + \int_0^{t-l} \chi^{\tilde{V}}(t,l,k)\bar{G}(k,l)dk - \tilde{M}(t,l)^2 \tag{S.107}$$

Now, the proof of overdispersion can begin. Firstly, it is helpful to bound $\tilde{M}(t,l)$ above, which can be done as follows. Squaring Supplementary Equation S.106 shows that

$$\tilde{M}(t,l)^2 = \bar{G}(t-l,l)^2\tilde{S}(t,l,t-l) + 2\bar{G}(t-l,l)\tilde{S}(t,l,t-l)^{0.5}\int_0^{t-l} \tilde{S}(t,l,u)^{0.5}g(u,l)du + \left[\int_0^{t-l} \tilde{S}(t,l,u)^{0.5}g(u,l)du\right]^2 \tag{S.108}$$

Now, using the Cauchy-Schwarz inequality, we see that

$$\left[\int_0^{t-l} \tilde{S}(t,l,u)^{0.5}g(u,l)du\right]^2 = \left[\int_0^{t-l} (\tilde{S}(t,l,u)g(u,l))^{0.5}(g(u,l))^{0.5}du\right]^2 \tag{S.109}$$

$$\leq \left[\int_0^{t-l} \tilde{S}(t,l,u)g(u,l)du\right]\left[\int_0^{t-l} g(u)du\right] \tag{S.110}$$

$$\leq (1-\bar{G}(t-l,l))\left[\int_0^{t-l} \tilde{S}(t,l,u)g(u,l)du\right] \tag{S.111}$$

Suppose that $\bar{G}(t-l,l) \neq 1$. Then, using

$$1 = \frac{1}{1-\bar{G}(t-l,l)} - \frac{\bar{G}(t-l,l)}{1-\bar{G}(t-l,l)} \tag{S.112}$$

to split the final term in Supplementary Equation S.108, we find

$$\tilde{M}(t,l)^2 \leq \bar{G}(t-l,l)^2\tilde{S}(t,l,t-l) + 2\bar{G}(t-l,l)\tilde{S}(t,l,t-l)^{0.5}\int_0^{t-l} \tilde{S}(t,l,u)^{0.5}g(u,l)du...$$

$$- \frac{\bar{G}(t-l,l)}{1-\bar{G}(t-l,l)}\left[\int_0^{t-l} \tilde{S}(t,l,u)^{0.5}g(u,l)du\right]^2 + \left[\int_0^{t-l} \tilde{S}(t,l,u)g(u,l)du\right] \tag{S.113}$$

To facilitate the remainder of this proof, it is helpful to define

$$Q(t,l) := \int_0^{t-l} \tilde{S}(t,l,u)^{0.5}g(u,l)du \tag{S.114}$$

Note that $Q(t,l) \geq 0$ as $\tilde{S}$ and g are non-negative. Moreover, for fixed t and l, the function $\tilde{S}(t,l,u)^{0.5}$ is non-decreasing in u and hence

$$Q(t,l) \leq \int_0^{t-l} \tilde{S}(t,l,t-l)^{0.5}g(u,l)du = \tilde{S}(t,l,t-l)^{0.5}(1-\bar{G}(t-l,l)) \tag{S.115}$$

Consider the function

$$f(Q) = 2\bar{G}(t-l,l)\tilde{S}(t,l,t-l)^{0.5}Q - \frac{\bar{G}(t-l,l)}{1-\bar{G}(t-l,l)}Q^2 \tag{S.116}$$

for $Q \in [0, \tilde{S}(t,l,t-l)^{0.5}(1-\bar{G}(t-l,l))]$. f is a quadratic, and has a single turning point at

$$f'(Q) = 0 \Rightarrow Q = \tilde{S}(t,l,t-l)^{0.5}(1-\bar{G}(t-l,l)) \tag{S.117}$$

This is an endpoint of the domain of Q and hence the maximum value of $f(Q)$ must occur one of the endpoints. $f(0) = 0$ and

$$f\Bigg(\tilde{S}(t,l,t-l)^{0.5}(1-\bar{G}(t-l,l))\Bigg) = \bar{G}(t-l,l)(1-\bar{G}(t-l,l))\tilde{S}(t,l,t-l) \tag{S.118}$$

This is non-negative, and hence the maximal value of $f(Q)$.

This can be put into the equation for $\tilde{M}(t,l)^2$ to give

$$\begin{aligned}\tilde{M}(t,l)^2 &\leq \bar{G}(t-l,l)^2\tilde{S}(t,l,t-l) + \bar{G}(t-l,l)(1-\bar{G}(t-l,l))\tilde{S}(t,l,t-l) + \left[\int_0^{t-l} S(t,l,u)g(u,l)du\right] \\ &= \bar{G}(t-l,l)\tilde{S}(t,l,t-l) + \left[\int_0^{t-l} S(t,l,u)g(u,l)du\right]\end{aligned} \tag{S.119}$$

Both the terms on the right hand side appear in the equation for $\tilde{V}$, and hence, substituting this result in shows that

$$\tilde{V}(t,l) \geq \int_0^{t-l} \chi^{\tilde{V}}(t,l,k)\bar{G}(k,l)dk \tag{S.120}$$

As this holds for all $\bar{G}(t-l,l) < 1$, it must also (under relevant continuity assumptions) hold for $\bar{G}(t-l,l) = 1$, and hence in all cases. Now,

$$\chi^{\tilde{V}}(t,l,k) = \tilde{V}(t,l+k)B(l+k)r(l+k,l) + \tilde{M}(t,l+k)^2\mathbb{E}(Y(l+k,l)^2)r(l+k,l) \tag{S.121}$$

As $Y \geq 1$ by definition, one has

$$B(l+k) = \mathbb{E}(Y(l+k,l)) \leq \mathbb{E}(Y(l+k,l)^2) \tag{S.122}$$

and hence

$$\chi^{\tilde{V}}(t,l,k) \leq \left[\tilde{V}(t,l+k)+\tilde{M}(t,l+k)^2\right]B(l+k)r(l+k,l) = \mathbb{E}(\tilde{Z}(t,l+k)^2)B(l+k)r(l+k,l) \tag{S.123}$$

Finally, as $\tilde{Z}(t, l+k) \geq 0$ and is integer-valued, one has $\tilde{Z}(t, l+k)^2 \geq \tilde{Z}(t, l+k)$ and hence

$$\chi^{\tilde{V}}(t, l, k) \leq \tilde{M}(t, l+k)B(l+k)r(l+k, l) \tag{S.124}$$

Thus,

$$\tilde{V}(t, l) \geq \int_0^{t-l} \tilde{M}(t, l+k)B(l+k)r(l+k, l)\bar{G}(k, l)dk = \tilde{M}(t, l+k) \tag{S.125}$$

which proves weak overdispersion.

To prove strict overdispersion, note that, for Supplementary Equation S.125 to hold to equality, it is necessary that all the inequalities used hold to equality. Thus, in particular, it is necessary that

$$\int_0^{t-l} \tilde{M}(t, l+k)B(l+k)r(l+k, l)\bar{G}(k, l)dk = \int_0^{t-l} \mathbb{E}(\tilde{Z}(t, l+k)^2)B(l+k)r(l+k, l)\bar{G}(k, l)dk \tag{S.126}$$

and hence, as $B(l+k) \geq 1$,

$$r(l+k, l)\bar{G}(k, l) \geq 0 \Rightarrow \mathbb{E}(\tilde{Z}(t, l+k)^2) = \tilde{M}(t, l+k) \tag{S.127}$$

This means that

$$r(l+k, l)\bar{G}(k, l) \geq 0 \Rightarrow \mathbb{E}(\tilde{Z}(t, l+k)(\tilde{Z}(t, l+k) - 1)) = 0 \tag{S.128}$$

and hence, as $\tilde{Z}(t, l+k)(\tilde{Z}(t, l+k) - 1)$ is a non-negative integer, this means that

$$r(l+k, l)\bar{G}(k, l) \geq 0 \Rightarrow \tilde{Z}(t, l+k)(\tilde{Z}(t, l+k) - 1) = 0 \tag{S.129}$$

almost surely. We now show that if $\mathbb{P}(\tilde{Z}(t, l) = 1) > 0$, then $\mathbb{P}(\tilde{Z}(t, l) > 1) > 0$. This can be done as follows.

Define the set $\mathcal{S}$ to be the possible times at which the initial individual can cause a secondary infection which in turn starts an epidemic that can have non-zero prevalence at time t. Then,

$$\mathcal{S} = \left\{ u \in (l, t-l) : r(l+u, l) > 0, \quad \bar{G}(u, l) > 0 \quad \text{and} \quad \mathbb{P}(Z(t, l+u) > 0) > 0 \right\} \tag{S.130}$$

Note the use of Z rather than $\tilde{Z}$. The first two conditions ensures that the likelihood of the initial individual causing an infection at time u is non-zero (as it must have non-zero rate here, and also

a non-zero probability of still being infectious). The third condition ensures that the probability of this secondary infection's infection tree still containing at least one infectious individual at time t is non-zero. It is necessary that

$$\int_{\mathcal{S}} r(l+u,l)\bar{G}(u,l)\mathbb{P}(Z(t,l+u)>0)du > 0 \tag{S.131}$$

as otherwise, $\tilde{Z}(t,l)=0$ (as this integral sums over all possible epidemics that lead to $\tilde{Z}(t,l)>0$). Define

$$\mathcal{S}(x) := \mathcal{S}\cap(l,x) \tag{S.132}$$

and the function

$$f(x) = \int_{\mathcal{S}(x)} r(l+u,l)\bar{G}(u,l)\mathbb{P}(Z(t,l+u)>0)du \tag{S.133}$$

Then, f must be continuous, and so there exists some $y\in(0,t-l)$ such that

$$0 < f(y) < f(t-l) = \int_{\mathcal{S}} r(l+u,l)\bar{G}(u,l)\mathbb{P}(Z(t,l+u)>0)du \tag{S.134}$$

Thus, there is a non-zero probability of an individual being infected in $(l,l+y)$ causing an epidemic that has non-zero prevalence at time t and, similarly, a non-zero probability of an individual being infected in $(l+y,t)$ causing an epidemic that has non-zero prevalence at time t. Thus, as the infections processes have independent increments and as the initial individual causing an infection in $(l+y,t)$ implies that it must have been infectious for the whole interval $(l,l+y)$, there is a non-zero probability of two such individuals being infected: one in $(l,l+y)$ and one in $(l+y,t)$. Hence

$$\mathbb{P}(\tilde{Z}(t,l)=1)>0 \Rightarrow \mathbb{P}(\tilde{Z}(t,l)>1)>0 \tag{S.135}$$

as required. Thus,

$$r(l+k,l)\bar{G}(k,l)\geq 0 \Rightarrow \tilde{Z}(t,l+k)=0 \tag{S.136}$$

and so

$$\mathbb{E}(\tilde{Z}(t,l+k))r(l+k,l)\bar{G}(k,l)=0 \quad \forall k \tag{S.137}$$

Thus, we have strict overdispersion, $\tilde{V}(t,l)>\tilde{M}(t,l)$, provided that the epidemic is expanded at time t, as required.

4.5 Comparison to a Poisson case

Consider comparing the variance Supplementary Equation S.91 with the variance of an epidemic where infection events are always of size 1 (that is, where the counting process of infections, $N^*(t,l)$ is a Poisson case, meaning $B^*(t) = 1$). Asterisks will be used to denote the quantities relating to this Poisson epidemic.

Suppose that the infectious period is the same in both cases (so $G = G^*$ and $\nu = \nu^*$). To ensure a fair comparison, it is also assumed that the mean number of cases is the same in both cases with $M(t,l) = M^*(t,l)$. By examining the Supplementary Equation S.72 for the mean, and absorbing κ into ρ in both cases, one can see

$$B(l+u)\rho(l+u) = \rho^*(l+u). \tag{S.138}$$

The variance Supplementary Equation S.91 can now be examined. Firstly, note that

$$\int_0^{t-l} M(t,l+u)B(l+u)r(l+u,l)du = \int_0^{t-l} M^*(t,l+u)r^*(l+u,l)du, \tag{S.139}$$

using the result above and the fact that $M(t,l+u) = M^*(t,l+u)$. Similarly,

$$S(t,l,u) = S^*(t,l,u). \tag{S.140}$$

Thus,

$$V(t,l) - V^*(t,l) = \int_0^{t-l} (\chi^V(t,l,k) - \chi^{V^*}(t,l,k))\bar{G}(k,l)dk \tag{S.141}$$

$$= \int_0^{t-l} \Big(V(t,l+k) - V^*(t,l+k)\Big)B(l+k)r(l+k,l)\bar{G}(k,l)dk... \tag{S.142}$$

$$+ \int_0^{t-l} \Big(\mathbb{E}(Y(l+k)^2) - 1\Big)M(t,l+k)^2 r(l+k,l)\bar{G}(k,l)dk \tag{S.143}$$

By defining $\Delta^V(t,l) := V(t,l) - V^*(t,l)$, one can see that this is a renewal equation

$$\Delta^V(t,l) = \int_0^{t-l} \Big(\mathbb{E}(Y(l+k)^2)-1\Big)M(t,l+k)^2 r(l+k,l)\bar{G}(k,l)dk + \int_0^{t-l} \Delta^V(t,l+k)B(l+k)r(l+k,l)\bar{G}(k,l)dk. \tag{S.144}$$

An important property of this renewal equation is that the part that is independent of Δ^V on the right hand side grows. That is,

$$\Delta^V(t,l) \geq \int_0^{t-l} \Big(\mathbb{E}(Y(l+k)^2) - 1\Big)M(t,l+k)^2 r(l+k,l)\bar{G}(k,l)dk. \tag{S.145}$$

Thus, even though these two epidemics give the same mean, the difference in their variances is proportional to the square of this mean. This means that models fitted to a Poisson process framework, even without exponential infectious periods, will substantially underestimate the variance of the number of cases (recalling that $\mathbb{E}(Y(l+k)^2) > 1$ in the non-Poisson case).

4.6 Large time solutions to the variance equation

To further understand the variance, we consider large time approximate solutions to the variance equation. Note that the level of rigour in this note is lower than the rest of our derivations as the results are derived for illustrative purposes.

It shall be assumed throughout this note that κ has been absorbed into ρ. Moreover, to enable explicit asymptotic solutions to be found, it shall be assumed that ρ, B and $\mathbb{E}(Y^2)$ are constants and that $g = g(t)$. Therefore all individuals behave identically (in distribution), irrespective of the time at which they were infected. Moreover, it means that $r(l+k, l) = r(k)$, as the rate of infection depends only on the time since the individual has been infected

Under these assumptions, the mean $M(t, l) = M(t-l)$ and the variance $V(t, l) = V(t-l)$ are functions of $t - l$ only. This property will be used when forming the heuristics used in this note.

The final assumption is that $\bar{G}(t)$ has a finite support - that is, $\bar{G}(t) = 0$ for sufficiently large t. This is not strictly necessary, but simplifies the analysis.

Then, for $t >> l$, the mean and variance equations become

$$M(t, l) = \int_0^{t-l} M(t, l+u) B\rho\nu(u)\bar{G}(u)du \tag{S.146}$$

and

$$V(t, l) = \int_0^{t-l} S(t, l, u)g(u, l) + \int_0^{t-l} \chi^V(t, l, k)\bar{G}(k)dk - M(t, l)^2. \tag{S.147}$$

Motivated by the exponential growth of epidemics without susceptible depletion, consider the heuristic

$$M(t, l) = e^{\gamma(t-l)} \tag{S.148}$$

for some growth rate γ (note that in Supplementary Equation S.146, scaling M by a constant does not affect the solution). Then, Supplementary Equation S.146 becomes

$$e^{\gamma(t-l)} = e^{\gamma(t-l)} \int_0^{t-l} e^{-\gamma u} B\rho\nu(u)\bar{G}(u)du. \tag{S.149}$$

Now, assuming that $t - l >> 1$, as the integrand has finite support,

$$e^{\gamma(t-l)} = e^{\gamma(t-l)} \int_0^{\infty} e^{-\gamma u} B\rho\nu(u)\bar{G}(u)du = e^{\gamma(t-l)} H(\gamma), \tag{S.150}$$

where $H(\gamma)$ is a monotonically decreasing function such that $H(-\infty) = \infty$ and $H(\infty) = 0$. It is necessary that

$$H(\gamma) = 1 \tag{S.151}$$

and, by the above notes on H, there is a unique value for γ (independent of l) such that this holds. We shall henceforth assume that γ is equal to this value.

Note that (by considering the case $\gamma = 0$)

$$\gamma > 0 \Leftrightarrow \int_0^{\infty} B\rho\nu(u)\bar{G}(u)du > 1 \tag{S.152}$$

and so the epidemic grows if and only if the expected number of cases caused by an individual is greater than 1, as expected.

The variance equation can now be considered. Note that

$$S(t, l, u) = \left[\int_0^u M(t, l + k)Br(k)dk\right]^2 = e^{2\gamma(t-l)} \left[\int_0^u e^{-\gamma k} Br(k)dk\right]^2. \tag{S.153}$$

Hence, the equation for the variance becomes

$$\begin{aligned} V(t, l) = e^{2\gamma(t-l)} \int_0^{t-l} \left[\int_0^u e^{-\gamma k} Br(k)dk\right]^2 g(u)du + \int_0^{t-l} V(t, l + k)Br(k)\bar{G}(k)dk... \\ + e^{2\gamma(t-l)} \int_0^{t-l} e^{-2\gamma k}\mathbb{E}(Y^2)r(k)\bar{G}(k)dk - e^{2\gamma(t-l)}. \end{aligned} \tag{S.154}$$

Note the χ^V term has been split into the two single integrals with integration variable k. This equation motivates a heuristic

$$V(t, l) = Ce^{2\gamma(t-l)}, \tag{S.155}$$

which, again using the fact that the integrands have finite support, results in

$$C = \frac{\int_0^\infty \left[\int_0^u e^{-\gamma k} Br(k) dk \right]^2 g(u) du + \int_0^\infty e^{-2\gamma k} \mathbb{E}(Y^2) r(k) \bar{G}(k) dk - 1}{1 - \int_0^\infty e^{-2\gamma k} Br(k) \bar{G}(k) dk}. \tag{S.156}$$

Note that

$$\int_0^\infty \left[\int_0^u e^{-\gamma k} Br(k) dk \right]^2 g(u) du > \int_0^\infty \left[\int_0^u e^{-\gamma k} Br(k) \bar{G}(k) dk \right]^2 g(u) du \tag{S.157}$$

$$= \int_0^\infty g(u) du \tag{S.158}$$

$$= 1. \tag{S.159}$$

and hence the numerator is strictly positive.

Moreover, suppose that $\gamma > 0$. Then, note that

$$\int_0^\infty e^{-2\gamma k} Br(k) \bar{G}(k) dk < \int_0^\infty e^{-\gamma k} Br(k) \bar{G}(k) dk = 1 \tag{S.160}$$

which means that the denominator (and hence C) is strictly positive.

Note that if $\gamma \leq 0$, this variance approximation is not well-defined (as C is either infinite if $\gamma = 0$ or negative if $\gamma < 0$) and so it is necessary to find another solution. In the $\gamma < 0$ case, $e^{\gamma(t-l)} >> e^{2\gamma(t-l)}$ and a leading-order solution can be found simply from

$$V = e^{\gamma(t-l)}. \tag{S.161}$$

Thus, according to these approximations, the variance grows with the square of the mean in the $\gamma > 0$ (i.e. growing epidemic) case, while it decreases proportionally to the mean in the $\gamma < 0$ (i.e. shrinking epidemic) case. The $\gamma = 0$ case is the bifurcation point between these two solutions and would require further analysis.

In the growing epidemic case, the equation for C is also informative in characterising the effect of the different model parameters on the variance. In particular, it shows that there is a linear relationship between $\mathbb{E}(Y(t)^2)$ and the variance, re-emphasising the point made in the previous subnote that ignoring this parameter can have significant effects on the variance estimate. Moreover, it shows that variance grows rapidly throughout a growing epidemic, remaining proportional to the square of the mean.

4.7 Mean and variance for cumulative incidence

The equations for the mean and prevalence of the cumulative incidence of the epidemic can be derived almost identically, as the two generating functions are very similar. The mean equation gains an term from the additional s being differentiated, which is

$$\int_0^{t-l} \mathcal{J}_{(l+u)}\Big(\int_0^{u} \mathcal{Y}_{(l+k,l)(1)}\frac{r(l+k,l)}{\lambda(l+u,l)}dk\Big)g(u,l)du = G(t-l,l) \tag{S.162}$$

and hence, the mean equation becomes (using *s to denote cumulative incidence quantities)

$$M^*(t,l) = 1 + \int_0^{t-l} M^*(t,l+u)\rho(l+u)\nu(u)\bar{G}(u,l)du \tag{S.163}$$

Now, the only difference in the equation for W in the case of cumulative incidence is that the term Supplementary Equation S.78 appears in both parts (again due to the extra s term). This can be treated in the same way as χ in the original derivation and so

$$\begin{aligned} W^*(t,l) = 2\int_0^{t-l} &\kappa M^*(t,l+u)B(l+u)r(l+u,l)\bar{G}(u,l)du + \bar{G}(t-l,l)\tilde{S}(t,l,t-l)... \\ ... + \int_0^{t-l} &\tilde{S}(t,l,u)g(u,l)du + \int_0^{t-l} \chi^*(t,l,k)\bar{G}(k,l)dk \end{aligned} \tag{S.164}$$

Again, following the previous derivation, one can then arrive at

$$\begin{aligned} V^*(t,l) = 1 + 2\int_0^{t-l} &\kappa M^*(t,l+u)B(l+u)r(l+u,l)\bar{G}(u,l)du + \bar{G}(t-l,l)\tilde{S}(t,l,t-l)... \\ ... + \int_0^{t-l} &\tilde{S}(t,l,u)g(u,l)du + \int_0^{t-l} \chi^{V^*}(t,l,k)\bar{G}(k,l)dk - M^*(t,l)^2 \end{aligned} \tag{S.165}$$

Supplementary Note: 5 Likelihood functions

5.1 Continuous case

If only the cumulative incidence, $Z(t,l)$, is known at some time t, the full epidemic history - in particular, the times at which each individual was infected, and the times at which they stopped being infectious - are unknown. Thus, it is helpful to derive a likelihood function for each possible

sequence of these times.

Perhaps the most intuitive approach would be to treat the times at which each individual was infected as continuous random variables. However, the resultant pdf is complicated by the fact that multiple infections are likely to happen simultaneously if $\mathbb{E}(Y) > 1$, and will have a significant number of Kronecker delta functions to accommodate this, making it complicated both mathematically and practically.

To remedy this, we instead consider three sets of random variables - a vector $\boldsymbol{T}$ of unknown size $n+1$, which contains the times of all the infection events up to time t; a vector $\boldsymbol{Y}$ also of size $n+1$, which contains the size of each of these infection events (that is, y_m is the number of individuals that are infected at time τ_m); and a vector $\boldsymbol{D}$ containing the times at which each individual stops being infected. To make the subsequent notation clearer, we shall use a non-rectangular array $\boldsymbol{X}$ in place of $\boldsymbol{D}$, where X_{ij} will be the time at which the jth individual infected at time T_i stops being infected.

We will suppose that for each $s > u$ and positive integer k

$$\mathbb{P}(N(s+dt,u) - N(s,u) = k) = p_k(s,u)dt + o(dt) \tag{S.166}$$

and that

$$\mathbb{P}(N(s+dt,u) - N(s,u) = 0) = 1 - \sum_{k\geq 1} p_k(s,u)dt + o(dt) = 1 - r(s,u)dt + o(dt) \tag{S.167}$$

as the counting process of jumps in $N(s,u)$ is an inhomogeneous Poisson Process of rate $r(s,u)$ (absorbing the κ into r). We can hence create a likelihood function. Define $\mathbf{1}$ to be a vector of 1's, and choose any vectors $\boldsymbol{\tau}$ and $\boldsymbol{d}$ such that each $\tau_i, d_j \in (0,t)$. Define dt to be small enough so that $\tau_i - \tau_j > dt$ for all $i > j$ and so that $|\tau_i - d_j| > dt$ for all i,j (note that, the set where $\tau_i = d_j$ has zero measure and can be ignored). Moreover, choose a positive-integer-valued vector $\boldsymbol{y}$. Then,

$$\begin{aligned}\mathbb{P}(\boldsymbol{T} \in [\boldsymbol{\tau}, \boldsymbol{\tau} + dt\mathbf{1}], \boldsymbol{D} \in [\boldsymbol{d}, \boldsymbol{d} + dt\mathbf{1}], \boldsymbol{Y} = \boldsymbol{y}) = P\Bigg[\Bigg(\bigcap_{k=1}^{n}\{y_k \text{ infections in } [\tau_k, \tau_k + dt]\}\Bigg)...\\ ... \cap \Bigg(\bigcap_{k=0}^{n}\{\text{no infections in } [\tau_k + dt, \tau_{k+1}]\Bigg) \cap \Bigg(\bigcap_{i=0}^{n}\bigcap_{j=1}^{y_i}\{L \in [x_{ij} - \tau_i, x_{ij} - \tau_i + dt]\}\Bigg)\Bigg]\end{aligned} \tag{S.168}$$

where $\tau_{n+1} := t$ to reduce notation, x_{ij} is the value of X_{ij} in the case $\boldsymbol{D} = \boldsymbol{d}$ and L is a random variable equal in distribution to the infectious period of an individual. Each of the infection events in the above equation occur on disjoint subintervals of $[0, t]$ and so, as all of the processes $N(t, l)$ have independent increments, and each individual behaves independently of each other and their infectious periods, they can be considered separately. We have

$$\mathbb{P}(y_k \text{ infections in } [\tau_k, \tau_k + dt]) = \sum_{i=0}^{k-1} \sum_{j=0}^{y_i} \mathbb{1}_{\{x_{ij} < \tau_k\}} p_{y_k}(\tau_k, \tau_i) dt + o(dt) \tag{S.169}$$

Here, the $o(dt)$ term contains three components that can be linearised out of the model - the probability that multiple different individuals contribute to the y_k cases (this is $O(dt^2)$); the probabilities of individuals infecting no one in this interval (these are independently $1 - O(dt)$ and hence the $O(dt)$ contribution can be ignored when these probabilities are multiplied together); and the $o(dt)$ terms from the equations defining p_k.

As the counting process of jumps in $N(s, u)$ is an inhomogeneous Poisson Process, and it is only "active" for individual ij up to time x_{ij},

$$\mathbb{P}(\text{no infections in } [\tau_k + dt, \tau_{k+1}]) = \prod_{i=0}^{k} \prod_{j=1}^{y_i} \exp\left(- \int_{\min(x_{ij}, \tau_k)}^{\min(x_{ij}, \tau_{k+1})} r(u, \tau_i) du \right) + O(dt) \tag{S.170}$$

where here, the $O(dt)$ term contains the integral between τ_k and $\tau_k + dt$ of each of the integrands. Taking the products inside the exponential as sums, the various "no infection" terms can be combined together to give

$$P\left(\bigcap_{k=0}^{n} \{\text{no infections in } [\tau_k + dt, \tau_{k+1}]\} \right) = \exp\left(- \sum_{i=0}^{n} \sum_{j=0}^{y_i} \int_{\tau_i}^{\min(t, x_{ij})} r(u, \tau_i) du \right) \tag{S.171}$$

Finally, the infectious period terms can be simply calculated from the pdf, g, of L as

$$\mathbb{P}(L \in [x_{ij} - \tau_i, x_{ij} - \tau_i + dt]) = g(x_{ij} - \tau_i, \tau_i) dt + o(dt) \tag{S.172}$$

Hence, combining all the relevant terms,

$$\mathbb{P}(T \in [\boldsymbol{\tau}, \boldsymbol{\tau} + dt\mathbf{1}], \boldsymbol{D} \in [\boldsymbol{d}, \boldsymbol{d} + dt\mathbf{1}], \boldsymbol{Y} = \boldsymbol{y}) = o(dt^{n+Z(t,l)}) +$$
$$\prod_{k=1}^{n} \left[\left(\prod_{j=1}^{y_k} g(x_{kj} - \tau_k, \tau_k) \right) \left(\sum_{i=0}^{k-1} \sum_{j=0}^{y_i} \mathbb{1}_{\{X_{ij} < \tau_k\}} p_{y_k}(\tau_k, \tau_i) \right) \right] \exp\left(- \sum_{i=0}^{n} \sum_{j=0}^{y_i} \int_{\tau_i}^{\min(t, X_{ij})} r(u, \tau_i) du \right) (dt)^{n+Z(t,l)} \tag{S.173}$$

and thus, taking $dt \to 0$ gives a likelihood function of

$$L(\boldsymbol{\tau}, \boldsymbol{y}, \boldsymbol{d}) = \prod_{k=1}^{n} \left[\left(\prod_{j=1}^{y_k} g(x_{kj} - \tau_k, \tau_k) \right) \left(\sum_{i=0}^{k-1} \sum_{j=0}^{y_i} \mathbb{1}_{\{x_{ij} < \tau_k\}} p_{y_k}(\tau_k, \tau_i) \right) \right] \exp\left(-\sum_{i=0}^{n} \sum_{j=0}^{y_i} \int_{\tau_i}^{\min(t, x_{ij})} r(u, \tau_i) du \right) \tag{S.174}$$

It is simple to substitute in the two examples that have been previously considered. In both cases, $r(a, b) = \rho(a)\nu(a - b)$. In the Poisson case, one has $p_1(a, b) = \rho(a)\nu(a - b)$ and $p_k(a, b) = 0$ for $k > 1$. In the Negative Binomial case, the values of p_k are given by

$$p_k(a, b)dt = \lim_{t \to 0} \left(\frac{\mathbb{P}(T \in [\boldsymbol{\tau}, \boldsymbol{\tau} + dt\mathbf{1}], \boldsymbol{D} \in [\boldsymbol{d}, \boldsymbol{d} + dt\mathbf{1}], \boldsymbol{Y} = \boldsymbol{y})}{dt^{n+Z(t,l)}} \right) \tag{S.175}$$

$$= \mathbb{P}(J_M(a + dt, b) - J_M(a, b) = 1)\mathbb{P}(Y = k) \tag{S.176}$$

$$= \rho(a)\nu(b - a) \left(\frac{(1 - p)^k}{-k \ln(p)} \right) \tag{S.177}$$

5.2 Special case (Poisson)

In the Poisson case, $A_{k,i}$ is Poisson distributed with mean $\rho(k)\nu(k - i)$. Hence,

$$\mathcal{A}_k(\boldsymbol{b}, \boldsymbol{y}, \boldsymbol{d}) \sim \text{Poi}\left(\rho(k) \sum_{i=0}^{k-1} \nu(k - i) \sum_{j=1}^{y_i} \mathbb{1}_{\{x_{ij} \le k\}} \right) := \text{Poi}(\mu_k) \tag{S.178}$$

and so, the more computationally useful log-likelihood is

$$\ell(\boldsymbol{\tau}, \boldsymbol{y}, \boldsymbol{D}) = \sum_{k=1}^{n} (\mu_k \log(y_k) - \mu_k - \log(y_k!)) + \sum_{i=1}^{n} \sum_{j=1}^{y_i} \log(g(x_{ij} - \tau_i, \tau_i)) \tag{S.179}$$

5.3 Special case (Negative Binomial)

In the Negative Binomial case,

$$A_{k,i} =_D \sum_{j=1}^{N} Y_j \tag{S.180}$$

where the Y_j are iid logarithmic random variables with a pmf given by Supplementary Equation S.44 that is independent of the properties of the individual, and N is Poisson distributed with mean $\rho(k)\nu(k - i)$. Thus,

$$\mathcal{A} \sim \mathcal{NB}(\phi\mu_k, p) \tag{S.181}$$

where, as before, $p = \frac{\phi}{1+\phi}$ and μ_k is defined in the previous note. Hence, as

$$\log\left[P\bigg(NB(a,p)=k\bigg)\right] = \sum_{j=0}^{k-1}\log(a+j) + k\log(1-p) + a\log(p) - \log(k!) \tag{S.182}$$

we have

$$\ell(\boldsymbol{\tau},\boldsymbol{y},\boldsymbol{D}) = \sum_{k=0}^{n}\left[\log(\phi\mu_k+j) + y_k\log\left(\frac{1}{1+\phi}\right) + \phi\mu_k\log\left(\frac{\phi}{1+\phi}\right) - \log(y_k!)\right] + \sum_{i=1}^{n}\sum_{j=1}^{y_i}\log(g(x_{ij}-\tau_i,\tau_i)) \tag{S.183}$$

5.4 Approximating the likelihood

It is difficult to simulate from the likelihoods when the infectious periods of the individuals are unknown because often, $Z(t,l) >> t$ (whereas the other unknowns, $\boldsymbol{\tau}$ and $\boldsymbol{y}$ have only $n \sim t$ parameters). To remedy this, we use an approximation - given an estimate of the function g, we simulate

$$D_i = L_i + \tau_i \quad \text{where } L_i \sim g \tag{S.184}$$

For some $\boldsymbol{D}$, the observed epidemic may be impossible (e.g. if, $D_0 < b_1$, where b_1 is the time that the first infection event occurs). Thus, it necessary to impose a feasibility condition. Many such conditions are possible, but we use a simple condition by defining

$$L_i^* := \max(L_i, \tau_{i+1} - \tau_i) \tag{S.185}$$

and then define

$$D_i^* := \tau_i + L_i^* \tag{S.186}$$

Given these values, we can then create an approximation, ℓ^* to be

$$\ell^*(\boldsymbol{\tau},\boldsymbol{y}) \sim \ell(\boldsymbol{\tau},\boldsymbol{y},\boldsymbol{D}^*) \tag{S.187}$$

This clearly creates a non-deterministic likelihood as it is dependent on a set of random variables. However, from our simulations, it appears that ℓ^* has a small variance, and so this extra randomness does not significantly affect our calculations.

Supplementary Note: 6 Assessing future variance during an epidemic

Many of the equations presented thus far have been concerned with properties of an epidemic started from a single case at a fixed deterministic time. However, it is crucial to be able to calculate the risk from any time during the epidemic, and such a derivation is presented in this note. This derivation is more algebraically involved than the other work in this paper, and so to reduce its length, it will be assumed that $N(t,l)$ is an inhomogeneous Poisson Process, and that $L=\infty$ for each individual. This means that $\boldsymbol{y}$ and $\boldsymbol{D}$ can be ignored when considering the likelihood.

6.1 Derivation

Suppose that the prevalence (or, equivalently in this case, cumulative incidence), $Z(t,l)=n+1$, is known at some point in an epidemic, but that the times at which these infections happened, B_i, are unknown. Note that the notation B_i rather than T_i is used in this note, because these times are now an exact analogue of birth times in a birth-death process. The condition of $n+1$ rather than n has been chosen as this means that there have been n new infections and will make the following derivation notationally simpler.

Note that the infection time of the initial individual, B_0 is known to be equal to l, but it will be treated identically to the other times to reduce notation. Its marginal pdf is $f_{B_0}(b)=\delta(b-l)$. Following the previous note, the pdf $f_{\boldsymbol{B}}(\boldsymbol{b})$ of the infection times is

$$f_{\boldsymbol{B}}(\boldsymbol{b})=\frac{1}{\mathbb{P}(Z(t,l)=n)}\prod_{i=1}^{n}\left(\rho(b_i)\sum_{j=0}^{i-1}\nu(b_i-b_j)\right)\exp\left[-\sum_{i=0}^{n}\int_0^{t-b_i}\rho(s+l)\nu(s)ds\right] \tag{S.188}$$

Now, one can write

$$Z(t+s,l)=\sum_{i=0}^{n}Z_i^*(t+s,B_i) \tag{S.189}$$

where $Z_i^*(t+s,B_i)$ counts the infection tree started at the individual infection at b_i, considering only those infections that occurred after time t (that is, if this individual infects someone at time

$a < t$, the infections of this second individual will *not* be counted, even if they occur after time t).

This can be rewritten as

$$Z(t+s,l) = \int_{b=0}^{t} \sum_{i=0}^{n} Z_i^*(t+s,b)\mathbb{1}_{\{B_i=b\}} \tag{S.190}$$

where here, $\mathbb{1}$ is the indicator function. Hence,

$$\begin{aligned} \mathrm{var}(Z(t+s,l)) &= \mathrm{var}\bigg(\int_{b=0}^{t} \sum_{i=0}^{n} Z_i^*(t+s,b)\mathbb{1}_{\{B_i=b\}} \bigg) && \text{(S.191)} \\ &= \int_{b=0}^{t} \sum_{i=0}^{n} \mathrm{var}(Z_i^*(t+s,b)\mathbb{1}_{\{B_i=b\}})... \\ &... + \int_{b=0}^{t}\int_{c=0}^{t} \sum_{i=0}^{n}\sum_{j=0}^{n} \mathrm{cov}\bigg(Z_i^*(t+s,b)\mathbb{1}_{\{B_i=b\}}, Z_j^*(t+s,b)\mathbb{1}_{\{B_j=c\}} \bigg)(\mathbb{1}_{\{(b,i)\neq(c,j)\}}) \\ & && \text{(S.192)} \end{aligned}$$

The first term in this equation can be expanded as

$$\begin{aligned} \mathrm{var}(Z_i^*(t+s,b)\mathbb{1}_{\{B_i=b\}}) &= \mathbb{E}(Z_i^*(t+s,b)^2\mathbb{1}^2_{\{B_i=b\}}) - \mathbb{E}(Z_i^*(t+s,b)\mathbb{1}_{\{B_i=b\}})^2 && \text{(S.193)} \\ &= \mathbb{E}(Z_i^*(t+s,b)^2)\mathbb{E}(\mathbb{1}_{\{B_i=b\}}) - \mathbb{E}(Z_i^*(t+s,b))^2\mathbb{E}(\mathbb{1}_{\{B_i=b\}})^2 && \text{(S.194)} \end{aligned}$$

Note that $\mathbb{E}(\mathbb{1}_{\{B_i=b\}})^2 = O(db^2)$ and hence this term has zero measure (as it is only integrated over one dimension). This leaves

$$\mathrm{var}(Z_i^*(t+s,b)\mathbb{1}_{\{B_i=b\}}) = \mathbb{E}(Z_i^*(t+s,b)^2)f_{B_i}(b)db \tag{S.195}$$

where $f_{B_i}(b)$ is the marginal pdf of B_i.

The second term can also be expanded - note that, by the independence of the Z^* terms, for $i \neq j$

$$\mathrm{cov}\bigg(Z_i^*(t+s,b)\mathbb{1}_{\{B_i=b\}}, Z_j^*(t+s,b)\mathbb{1}_{\{B_j=c\}} \bigg) = \mathbb{E}(Z_i^*(t+s,b))\mathbb{E}(Z_j^*(t+s,c))\mathrm{cov}(\mathbb{1}_{\{B_i=b\}}, \mathbb{1}_{\{B_j=c\}}) \tag{S.196}$$

Moreover, if $i = j$, then one has $b \neq c$ and hence

$$\mathbb{1}_{\{B_i=b\}}\mathbb{1}_{\{B_j=c\}} = \mathbb{1}_{\{B_i=b,B_i=c\}} = 0 \tag{S.197}$$

which means

$$\mathrm{cov}\bigg(Z_i^*(t+s,b)\mathbb{1}_{\{B_i=b\}},Z_j^*(t+s,b)\mathbb{1}_{\{B_j=c\}}\bigg) = -\mathbb{E}(Z_i^*(t+s,b))\mathbb{E}(Z_j^*(t+s,c))\mathbb{E}(\mathbb{1}_{\{B_i=b\}})\mathbb{E}(\mathbb{1}_{\{B_j=c\}}) \tag{S.198}$$

$$= \mathbb{E}(Z_i^*(t+s,b))\mathbb{E}(Z_j^*(t+s,c))\mathrm{cov}(\mathbb{1}_{\{B_i=b\}},\mathbb{1}_{\{B_j=c\}}) \tag{S.199}$$

and hence the Supplementary Equation S.196 holds in all cases. Now, one has, for $i \neq j$

$$\mathrm{cov}(\mathbb{1}_{\{B_i=b\}},\mathbb{1}_{\{B_j=c\}}) = \mathbb{E}(\mathbb{1}_{\{B_i=b\}}\mathbb{1}_{\{B_j=c\}}) - \mathbb{E}(\mathbb{1}_{\{B_i=b\}})\mathbb{E}(\mathbb{1}_{\{B_j=c\}}) \tag{S.200}$$

$$= \mathbb{E}(\mathbb{1}_{\{B_i=b,B_j=c\}}) - f_{B_i}(b)f_{B_j}(c)dbdc \tag{S.201}$$

$$= (f_{B_i,B_j}(b,c) - f_{B_i}(b)f_{B_j}(c))dbdc \tag{S.202}$$

while if $i = j$ and $b \neq c$, this result also holds, following the convention that

$$f_{B_i,B_i}(b,c) = \delta(b-c)f_{B_i}(b) \tag{S.203}$$

(and hence in this case is zero) where δ is the Kronecker delta.

Thus, in all cases

$$\mathrm{cov}\bigg(Z_i^*(t+s,b)\mathbb{1}_{\{B_i=b\}},Z_j^*(t+s,b)\mathbb{1}_{\{B_j=c\}}\bigg) = \mathbb{E}(Z_i^*(t+s,b))\mathbb{E}(Z_j^*(t+s,c))(f_{B_i,B_j}(b,c)-f_{B_i}(b)f_{B_j}(c))dbdc \tag{S.204}$$

This gives an equation of

$$\begin{aligned}\mathrm{var}(Z(t+s,l)) = &\int_{b=0}^{t}\sum_{i=0}^{n}\mathbb{E}(Z_i^*(t+s,b)^2)f_{B_i}(b)db...\\ ... + &\int_{b=0}^{t}\int_{c=0}^{t}\sum_{i=0}^{n}\sum_{j=0}^{n}\mathbb{E}(Z_i^*(t+s,b))\mathbb{E}(Z_j^*(t+s,c))(f_{B_i,B_j}(b,c)-f_{B_i}(b)f_{B_j}(c))\mathbb{1}_{\{(b,i)\neq(c,j)\}}dbdc\end{aligned} \tag{S.205}$$

It is more informative to remove the $\mathbb{1}_{\{(b,i)\neq(c,j)\}}$ condition. This can be done by calculating

$$\int_{b=0}^{t}\int_{c=0}^{t}\sum_{i=0}^{n}\sum_{j=0}^{n}\mathbb{E}(Z_i^*(t+s,b))\mathbb{E}(Z_j^*(t+s,c))\Big(f_{B_i,B_j}(b,c)-f_{B_i}(b)f_{B_j}(c)\Big)\mathbb{1}_{\{(b,i)=(c,j)\}}dbdc \tag{S.206}$$

$$=\int_{b=0}^{t}\int_{c=0}^{t}\sum_{i=0}^{n}\mathbb{E}(Z_i^*(t+s,b)\mathbb{E}(Z_i^*(t+s,c))\Big(\delta(b-c)f_{B_i}(b)-f_{B_i}(b)f_{B_i}(c)\Big)\mathbb{1}_{\{b=c\}}dbdc \tag{S.207}$$

$$=\int_{b=0}^{t}\int_{c=0}^{t}\sum_{i=0}^{n}\mathbb{E}(Z_i^*(t+s,b)\mathbb{E}(Z_i^*(t+s,c))\Big(\delta(b-c)f_{B_i}(b)-f_{B_i}(b)f_{B_i}(c)\mathbb{1}_{\{b=c\}}\Big)dbdc \tag{S.208}$$

$$=\int_{b=0}^{t}\sum_{i=0}^{n}\mathbb{E}(Z_i^*(t+s,b))^2 f_{B_i}(b)db \tag{S.209}$$

noting that the second term is bounded and contains $\mathbb{1}_{\{b=c\}}$ which is non-zero only on a null set of the domain of integration (and hence the integral is zero). Thus, absorbing this correction term into the first term in Supplementary Equation S.205,

$$\mathrm{var}(Z(t+s,l))=\int_{b=0}^{t}\sum_{i=0}^{n}\mathrm{var}(Z_i^*(t+s,b))f_{B_i}(b)db...$$

$$...+\int_{b=0}^{t}\int_{c=0}^{t}\sum_{i=0}^{n}\sum_{j=0}^{n}\mathbb{E}(Z_i^*(t+s,b))\mathbb{E}(Z_j^*(t+s,c))(f_{B_i,B_j}(b,c)-f_{B_i}(b)f_{B_j}(c))dbdc \tag{S.210}$$

The advantage of this formulation is that it allows the contributions to the variance from the infection times B_i before time t and from further infections between times t and $t+s$ to be separated. Indeed, note that if the infection times are known (so that $f_{B_i}(b)=\delta(b-b_i)$), one has

$$\int_{b=0}^{t}\int_{c=0}^{t}\sum_{i=0}^{n}\sum_{j=0}^{n}\mathbb{E}(Z_i^*(t+s,b))\mathbb{E}(Z_j^*(t+s,c))(f_{B_i,B_j}(b,c)-f_{B_i}(b)f_{B_j}(c))dbdc$$

$$...=\int_{b=0}^{t}\int_{c=0}^{t}\sum_{i=0}^{n}\sum_{j=0}^{n}\mathbb{E}(Z_i^*(t+s,b))\mathbb{E}(Z_j^*(t+s,c))(\delta(b-b_i)\delta(c-b_j)-\delta(b-b_i)\delta(c-b_j))dbdc \tag{S.211}$$

$$=0 \tag{S.212}$$

noting that the definition of

$$f_{B_i,B_i}(b,c)=f_{B_i}(b)\delta(b-c)=f_{B_i,B_i}(b,c)=\delta(b-b_i)\delta(b-c)=\delta(b-b_i)\delta(c-b_i) \tag{S.213}$$

is consistent in this case. Thus, the second term in Supplementary Equation S.210 is only non-zero when there is uncertainty in the infection times (while, moreover, the first term is only non-

zero when there is uncertainty in the infections that occur in the interval $(t, t+s)$, as otherwise $\text{var}(Z_i^*(t+s, b_i)) = 0)$.

To complete the derivation of the variance equation, it is necessary to derive formulae to calculate the quantities $\text{var}(Z_i^*)$. To enable this, define $M^*(t+s, b_i) := \mathbb{E}(Z_i^*(t+s, b_i))$ and $X^*(t+s, b_i) := \mathbb{E}(Z_i^*(t+s, b_i)^2)$ to be the mean and squared mean of the infection tree started from time t by the ith individual.

These quantities can be calculated directly from the mean and variance, M and V, of the "standard case" (where a single initial individual is infected at some time l), considered in previous notes in this appendix. This is possible as, in the context of renewal processes, the quantities Z_i^* are renewal processes where all but the first individuals are identical, and hence are amenable to similar methodology. Indeed, if one supposes that $\{Z(t+s, t+u)\}_{u \leq s}$ are a set of independent realisations of different "standard" epidemics, one has

$$Z_i^*(t+s, t+u) = \int_{u=0}^{s} Z(t+s, t+u) \mathbb{1}_{\{\text{individual } i \text{ infects another individual at time } t+u\}} \tag{S.214}$$

as the newly infected individuals start new, independent and "standard" epidemics. Define

$$\mathcal{I}_u := \mathbb{1}_{\{\text{individual } i \text{ infects another individual at time } t+u\}} \tag{S.215}$$

Hence,

$$M^*(t+s, b_i) = E\bigg(\int_{u=0}^{s} Z(t+s, t+u)\mathcal{I}_u\bigg) \tag{S.216}$$

$$= \int_{u=0}^{s} M(t+s, t+u)\rho(t+u)\nu(t-b_i+u)du \tag{S.217}$$

Moreover,

$$X^*(t+s, b_i) = E\bigg(\bigg[\int_{u=0}^{s} Z(t+s, t+u)\mathcal{I}_u\bigg]^2\bigg) \tag{S.218}$$

$$= E\bigg(\int_{u=0}^{s}\int_{k=0}^{s} Z(t+s, t+u)\mathcal{I}_u Z(t+s, t+k)\mathcal{I}_k\bigg) \tag{S.219}$$

Note that, for $k \neq u$, the quantities $Z(t+s, t+u)$ and $Z(t+s, t+k)$ are independent. Moreover, these quantities are all independent from the indicator terms. Thus, it is helpful to split the

integral, giving

$$W^*(t+s,b_i) = \int_{u=0}^{s} E\Big(Z(t+s,t+u)^2\mathcal{I}_u\Big) + \int_{u=0}^{s}\int_{k=0}^{s} E\Big[Z(t+s,t+u)\mathcal{I}_u Z(t+s,t+k)\mathcal{I}_k\Big]\mathbb{1}_{\{u\neq k\}} \tag{S.220}$$

$$= \int_{u=0}^{s} E\Big(Z(t+s,t+u)^2\mathcal{I}_u\Big) + \int_{u=0}^{s}\int_{k=0}^{s} M(t+s,t+u)\mathbb{E}(\mathcal{I}_u)M(t+s,t+k)\mathbb{E}(\mathcal{I}_k)\mathbb{1}_{\{u\neq k\}} \tag{S.221}$$

Now,

$$\int_{u=0}^{s}\int_{k=0}^{s} M(t+s,t+u)\mathbb{E}(\mathcal{I}_u)M(t+s,t+k)\mathbb{E}(\mathcal{I}_k) = \Big[\int_{u=0}^{s} M(t+s,t+u)\mathbb{E}(\mathcal{I}_u)\Big]^2 = M^*(t+s,b_i)^2 \tag{S.222}$$

while

$$\int_{u=0}^{s}\int_{k=0}^{s} M(t+s,t+u)\mathbb{E}(\mathcal{I}_u)M(t+s,t+k)\mathbb{E}(\mathcal{I}_k)\mathbb{1}_{\{u=k\}} = 0 \tag{S.223}$$

as the integrand is bounded and is non-zero only on a null set of the domain of integration. Hence, one has

$$\int_{u=0}^{s}\int_{k=0}^{s} M(t+s,t+u)\mathbb{E}(\mathcal{I}_u)M(t+s,t+k)\mathbb{E}(\mathcal{I}_k)\mathbb{1}_{\{u\neq k} = M^*(t+s,b_i)^2 \tag{S.224}$$

Thus,

$$X^*(t+s,b_i) = \int_{u=0}^{s} E\Big(Z(t+s,t+u)^2\mathcal{I}_u\Big) + M^*(t+s,b_i)^2 \tag{S.225}$$

$$= \int_{u=0}^{s} E\Big(Z(t+s,t+u)^2\Big)\mathbb{E}(\mathcal{I}_u) + M^*(t+s,b_i)^2 \tag{S.226}$$

$$= \int_{u=0}^{s} (V(t+s,t+u) + M(t+s,t+u)^2)\rho(t+u)\nu(t-b_i+u)du + M^*(t+s,b_i)^2 \tag{S.227}$$

Hence, defining $V^*(t+s,b_i) := \text{var}(Z^*(t+s,b_i)) = X^*(t+s,b_i) - M^*(t+s,b_i)^2$, one has

$$V^*(t+s,b_i) = \int_{u=0}^{s} (V(t+s,t+u) + M(t+s,t+u)^2)\rho(t+u)\nu(t-b_i+u)du \tag{S.228}$$

Hence, one has the final form of the variance equation

$$\text{var}(Z(t+s,l)) = \int_{b=0}^{t}\sum_{i=0}^{n} V^*(t+s,b)f_{B_i}(b)db...$$

$$... + \int_{b=0}^{t}\int_{c=0}^{t}\sum_{i=0}^{n}\sum_{j=0}^{n} M^*(t+s,b)M^*(t+s,c)(f_{B_i,B_j}(b,c) - f_{B_i}(b)f_{B_j}(c))dbdc \tag{S.229}$$

6.2 Bounding the equation

Unlike previous formulae, this is an explicit equation and no recursion is required to get the desired results (although recursion is necessary to calculate the V term in V^*). However, the infection time pdf makes this a difficult equation to evaluate.

However, one can give a simpler upper bound on the variance. Define

$$\nu_{\mathrm{bound}}(u) := \max_{b_i \in [l,t]} (\nu(t - b_i + u)) \tag{S.230}$$

so that

$$M^*(t+s, b_i) \leq \int_0^s M(t+s, t+u)\rho(t+u)\nu_{\mathrm{bound}}(t - b_i + u)du := \mathcal{M}^*(t+s) \tag{S.231}$$

and

$$V^*(t+s, b_i) \leq \int_{u=0}^s (V(t+s, t+u) + M(t+s, t+u)^2)\rho(t+u)\nu_{\mathrm{bound}}(u)du := \mathcal{V}^*(t+s) \tag{S.232}$$

so that this is now independent of b_i. Note that the construction of $\nu_{\mathrm{bound}}(u)$ means that it will still decay for large u. Under the assumption that the infection times are roughly deterministic so the second term is zero,

$$\text{var } (Z(t+s, l)) \leq Z(t, l)\mathcal{V}^*(t+s) \tag{S.233}$$

The covariance term can be added in by noting that

$$\int_{b=0}^t \int_{c=0}^t \sum_{i=0}^n \sum_{j=0}^n M^*(t+s, b)M^*(t+s, c)(f_{B_i,B_j}(b,c) - f_{B_i}(b)f_{B_j}(c))dbdc...$$

$$... \leq \int_{b=0}^t \int_{c=0}^t \sum_{i=0}^n \sum_{j=0}^n \mathcal{M}^*(t+s)^2 f_{B_i,B_j}(b,c)dbdc \tag{S.234}$$

$$\leq \sum_{i=0}^n \sum_{j=0}^n \int_{b=0}^t \int_{c=0}^t \mathcal{M}^*(t+s)^2 (f_{B_i,B_j}(b,c) + f_{B_i}(b)f_{B_j}(c))dbdc \tag{S.235}$$

$$\leq Z(t, l)^2 \mathcal{M}^*(t+s)^2 \tag{S.236}$$

which gives an overall bound of

$$\text{var } (Z(t+s, l)) \leq Z(t, l)\mathcal{V}^*(t+s) + Z(t, l)^2 \mathcal{M}^*(t+s)^2 \tag{S.237}$$

6.3 Special cases

To finish, it is helpful to consider a couple of special cases which may arise when the epidemic is large. If the infection times are mostly independent, then

$$i \neq j \Rightarrow f_{B_i,B_j}(b,c) \sim f_{B_i}(b) f_{B_j}(c) \tag{S.238}$$

while for $i = j$, note that

$$\int_{b=0}^{t}\int_{c=0}^{t}\sum_{i=0}^{n} M^*(t+s,b)M^*(t+s,c)(f_{B_i,B_i}(b,c) - f_{B_i}(b)f_{B_i}(c))dbdc...$$

$$... = \int_{b=0}^{t}\int_{c=0}^{t}\sum_{i=0}^{n} M^*(t+s,b)M^*(t+s,c)(\delta(b-c)f_{B_i}(b) - f_{B_i}(b)f_{B_i}(c))dbdc \tag{S.239}$$

$$= \int_{b=0}^{t}\sum_{i=0}^{n} M^*(t+s,b)^2 f_{B_i}(b)db - \sum_{i=0}^{n}\left[\int_b M^*(t+s,b)f_{B_i}(b)db\right]^2 \tag{S.240}$$

and hence

$$\mathrm{var}(Z(t+s,l)) \sim \int_{b=0}^{t}\sum_{i=0}^{n} V^*(t+s,b)f_{B_i}(b)db + \int_b \sum_{i=0}^{n} M^*(t+s,b)^2 f_{B_i}(b)db - \sum_{i=0}^{n}\left[\int_b M^*(t+s,b)f_{B_i}(b)db\right]^2 \tag{S.241}$$

This is still a complicated equation to compute, although the advantage is that one only needs one-dimensional marginal distributions of the infection times, and hence it is significantly more tractable. Moreover, the upper bound on the variance can be improved to

$$\mathrm{var}(Z(t+s,l)) \leq Z(t,l)\mathcal{V}(t,l) + Z(t,l)\mathcal{M}(t,l)^2 \tag{S.242}$$

so that it is proportional to $Z(t,l)$, rather than $Z(t,l)^2$.

The simplest case is when the infection times are known - something which may be approximately true if the epidemic is large (and hence has been approximately deterministic in the recent past). In this case, the equation simply reduces to

$$\mathrm{var}(Z(t+s,l)) \sim \sum_{i=0}^{n} V^*(t+s,b_i) \tag{S.243}$$

where b_i are the infection times. In this case, the variance can be simply calculated from the quantities M and V.

Supplementary Note: 7 Discrete epidemics

7.1 Discrete pgf

Suppose now that the branching process is entirely discrete (and, for convenience, occurs on integer times). For the lifetime, L, of an individual infected at l, define

$$g(u,l) := \mathbb{P}(L = u) \quad \text{and} \quad \overline{G}(u,l) := \mathbb{P}(L \geq u) \tag{S.244}$$

In this discrete setting, it is important to specify exactly inequalities whose strictness is unimportant in the continuous case. In particular, if an individual is infected at time a and has a lifetime of b, it will be considered to be infectious at time $a+b$, and will be counted when calculating prevalence at this time. That is, it can infect others at time $a+b$ (and these individuals will be given infection time $a+b$) but will not be able to infect individuals at time $a+b+1$.

For the counting process of infections, one can in this case work without a separate infection event process and instead simply use the quantities

$$q_u(t,l) := \mathbb{P}\Big(N(t,l) - N(t-1,l) = u\Big) \quad \text{and} \quad \mathcal{Q}_{(t,l)}(s) := E\Big(s^{Q(t,l)}\Big) \tag{S.245}$$

where $Q(t,l)$ has pmf given by $q_u(t,l)$. Hence, each $Q(t,l)$ (which may be zero, unlike Y in the continuous case) gives the number of new infections at time t caused by an individual that was infected at time l. Now, note that for $u < t-l$, one has

$$E\Big(s^{Z(t,l)}\Big| L = u\Big) = E\Big(s^{\sum_{k=1}^{u}\sum_{i=1}^{Q(l+k,l)} Z_{ik}(l+u,l)}\Big) \tag{S.246}$$

where the variables Z_{ik} are iid copies of Z. Note that the variables $Q(l+k,l)$ are independent as $N(t,l)$ has indepedent increments, meaning that

$$E\Big(s^{Z(t,l)}\Big| L = u\Big) = \prod_{k=1}^{u} E\Big(s^{\sum_{i=1}^{q(l+k,l)} Z_{ik}(l+u,l+k)}\Big) \tag{S.247}$$

$$= \prod_{k=1}^{u} \mathcal{Q}_{(l+k,l)}\Big(F(l+u,l+k)\Big) \tag{S.248}$$

Thus, the generating function equation for prevalence can be written as

$$F(t,l) = s\overline{G}(t-l,l)\prod_{k=1}^{t-l}\mathcal{Q}_{(l+k,l)}\Big(F(t,l+k)\Big) + \sum_{u=0}^{t} g_u \prod_{k=1}^{u}\mathcal{Q}_{(l+k,l)}\Big(F(t,l+k)\Big) \tag{S.249}$$

where

$$\overline{G}(t-l,l) = \mathbb{P}(L \geq t-l) \tag{S.250}$$

The form of the generating function for the discrete case is simpler than the continuous one and might be more amenable to computation.

7.2 Recovery of the continuous case

Suppose that each step corresponds to a time interval of $dt << 1$. Suppose further that

$$\hat{g}(udt, ldt)dt \sim g_{u,l}, \quad \hat{t} \sim tdt, \quad \text{and} \quad \hat{l} \sim ldt \tag{S.251}$$

where the quantities with a hat are constant. To ensure continuity in probability, it will be assumed that

$$\hat{q}_u(\hat{t},\hat{l})dt \sim q_u(t,l) \quad \forall u \geq 1 \quad \text{and} \quad q_0(t,l) \sim 1 - \sum_{u=1}^{\infty} dt\hat{q}_u(\hat{t},\hat{l}) \tag{S.252}$$

where again, $\hat{q}$ is independent of dt. Now, one has

$$G(t-l,l) = \sum_{u=0}^{t-l} g_{u,l} \sim \sum_{u=0}^{\frac{\hat{t}-\hat{l}}{dt}} \hat{g}_{u,l}(udt)dt \sim \int_0^{\hat{t}-\hat{l}} \hat{g}(u,\hat{l})du := \hat{G}(\hat{t}-\hat{l},l) \tag{S.253}$$

Moreover, one has

$$\mathcal{Q}_{(t,l)}(s) \sim \left(1 - \sum_{u=1}^{\infty} \hat{q}_u(\hat{t},\hat{l})dt\right) + \sum_{u=1}^{\infty} s^u \hat{q}_u(\hat{t},\hat{l})dt = 1 + \sum_{u=1}^{\infty}(s^u - 1)\hat{q}_u(\hat{t},\hat{l})dt \tag{S.254}$$

Using this relation, setting $\hat{k} := kdt$ and Taylor expanding gives

$$\log\left(\prod_{k=1}^{t-l} \mathcal{Q}_{(l+k,l)}(s)\right) \sim \sum_{k=1}^{t-l} \log\left(1 + \sum_{u=1}^{\infty}(s^u-1)\hat{q}_u(\hat{l}+\hat{k},\hat{l})dt\right) \tag{S.255}$$

$$\sim \sum_{k=1}^{t-l}\sum_{u=1}^{\infty}(s^u-1)\hat{q}_u(\hat{l}+\hat{k},\hat{l})dt \tag{S.256}$$

$$\sim \int_0^{\hat{t}-\hat{l}} \sum_{u=1}^{\infty}(s^u-1)\hat{q}_u(\hat{l}+\hat{k},\hat{l})d\hat{k} \tag{S.257}$$

Hence,

$$F(t,l) \sim (1-\hat{G}(\hat{t}-\hat{l}))\exp\left[\int_0^{\hat{t}-\hat{l}} \sum_{u=1}^{\infty}(s^u-1)\hat{q}_u(\hat{l}+\hat{k},\hat{l})d\hat{k}\right] + \int_0^{t-l} \exp\left[\int_0^{\hat{u}} \sum_{w=1}^{\infty}(s^w-1)\hat{q}_w(\hat{l}+\hat{k},\hat{l})d\hat{k}\right]\hat{g}(\hat{u},\hat{l})d\hat{u} \tag{S.258}$$

It is now possible to define the limiting continuous process. Consider a counting process $N(\hat{t},\hat{l})$ in continuous time with independent increments where infection events occur according to a rate function given by

$$r(\hat{t},\hat{l}) = \sum_{u=1}^{\infty} \hat{q}_u(\hat{t},\hat{l}) \tag{S.259}$$

and where, given that a infection event occurs at t from a particle born at l, the infection event is of size $k \geq 0$ with probability

$$\frac{\hat{q}_k(\hat{t},\hat{l})}{\sum_{u=1}^{\infty} \hat{q}_u(\hat{t},\hat{l})}. \tag{S.260}$$

Suppose that $J(\hat{t},\hat{l})$ counts the infection events of this process (and hence is an inhomogeneous Poisson Process of rate $r(\hat{t},\hat{l})$) and that $\mathcal{Y}_{(\hat{t},\hat{l})}$ is the generating function of infection event size given that a infection event occurs at $(\hat{t},\hat{l})$. Note that

$$\int_0^{\hat{t}-\hat{l}} \sum_{u=1}^{\infty} \hat{q}_u(\hat{l}+k,\hat{l})dk = \int_0^{\hat{t}-\hat{l}} r(\hat{l}+k,\hat{l})dk = \mathbb{E}(J(\hat{t},\hat{l})) \tag{S.261}$$

and that

$$\sum_{u=1}^{\infty} s^u \hat{q}_u(\hat{l}+\hat{k},\hat{l}) = \sum_{u=1}^{\infty} \left(\frac{s^u \hat{y}_u(\hat{l}+\hat{k},\hat{l})}{\sum_{m=1}^{\infty} \hat{y}_m(\hat{l}+\hat{k},\hat{l})} \right) \sum_{m=1}^{\infty} \hat{q}_m(\hat{l}+\hat{k},\hat{l}) \tag{S.262}$$

$$= \mathcal{Y}_{(\hat{l}+\hat{k},\hat{l})}(s) \left(\sum_{u=1}^{\infty} \hat{q}_u(\hat{l}+\hat{k},\hat{l}) \right) \tag{S.263}$$

$$= \mathcal{Y}_{(\hat{l}+\hat{k},\hat{l})}(s) r(\hat{l}+\hat{k},\hat{l}) \tag{S.264}$$

Hence,

$$\prod_{k=1}^{t-l} \mathcal{Q}_{(l+k,l)}(s) \sim \exp\left[\int_0^{\hat{t}-\hat{l}} r(\hat{l}+k,\hat{l}) \mathcal{Y}_{(\hat{l}+k,\hat{l})}(s)dk - \mathbb{E}(J(\hat{t},\hat{l})) \right] \tag{S.265}$$

and so, applying this to Supplementary Equation S.258 shows that the continuous generating function equation is recovered. Note that here, the distribution of Y has been allowed to depend on l (and this is the generating function equation that arises in this case), but the an equation with an l-independent Y will arise if the ratio of each $q_k(t,l)$ and $\sum_{k=1}^{\infty} q_k(t,l)$ are independent of l.

7.3 Distinctness from the continuous case

It is important to note that the relaxation of the assumption that N is continuous in probability necessary in considering the discrete case means that the pgf becomes materially different.

Indeed, one can characterise the discrete case through the continuous framework by imposing that

$$r(t,l) = \Big(\sum_{u=1}^{\infty} q_u(t,l)\Big)\Big(\sum_{n=1}^{\infty} \delta(l+n-t)\Big) \tag{S.266}$$

as this is gives probability of N increasing (by whatever number) in the discrete case discussed above. Moreover, again allowing Y to depend on l, $Y(t,l)$ has distribution

$$\mathbb{P}(Y(t,l)=k) = \frac{q_k(t,l)}{\sum_{m=1}^{\infty} q_m(t,l)} \tag{S.267}$$

Now, note that

$$\lambda(t,l) = \int_l^t r(s,l)ds = \sum_{n=1}^{\lfloor t-l \rfloor}\sum_{u=1}^{\infty} q_u(l+n,l) \tag{S.268}$$

where $\lfloor m \rfloor$ denotes the largest integer that is smaller than m. Moreover

$$\int_0^{t-l} \mathcal{Y}_{(l+k,l)}(F(t,l+k))r(l+k,l) = \sum_{n=1}^{\lfloor t-l \rfloor}\sum_{u=1}^{\infty} q_u(l+n,l)\mathcal{Y}_{(l+n,l)}(F(t,l+n)) \tag{S.269}$$

We suppose for a contradiction that the pgf in the continuous case is also valid in this discrete setting. Hence (taking $\kappa = 1$)

$$F(t,l) = s(1-G(t-l,l))\exp\Bigg[\sum_{n=1}^{\lfloor t-l \rfloor}\sum_{u=1}^{\infty} q_u(l+n,l)\mathcal{Y}_{(l+n,l)}(F(t,l+n)) - \sum_{n=1}^{\lfloor t-l \rfloor}\sum_{u=1}^{\infty} q_u(l+n,l)\Bigg]...$$
$$... + \int_0^{t-l} \exp\Bigg[\sum_{n=1}^{\lfloor t-l+u \rfloor}\sum_{m=1}^{\infty} q_m(l+n,l)\mathcal{Y}_{(l+n,l)}(F(t,l+n)) - \sum_{n=1}^{\lfloor t-l+u \rfloor}\sum_{m=1}^{\infty} q_m(l+n,l)\Bigg] g(u,l)du \tag{S.270}$$

Now, note that

$$\mathcal{Y}_{(l+n,l)}(s) = \sum_{m=1}^{\infty} \frac{s^m q_m(l+n,l)}{\sum_{k=1}^{\infty} q_k(l+n,l)} \tag{S.271}$$

$$= \frac{1}{\sum_{k=1}^{\infty} q_k(l+n,l)}\Big(\sum_{m=0}^{\infty} s^m q_m(l+n,l) - q_0(l+n,l)\Big) \tag{S.272}$$

$$= \frac{1}{\sum_{k=1}^{\infty} q_k(l+n,l)}\Big(\mathcal{Q}_{(l+n,l)}(s) - (1-\sum_{k=1}^{\infty} q_k(l+n,l))\Big) \tag{S.273}$$

and hence

$$\sum_{n=1}^{\lfloor t-l+u \rfloor} \sum_{m=1}^{\infty} q_m(l+n,l)\mathcal{Y}_{(l+n,l)}(F(t,l+n)) - \sum_{n=1}^{\lfloor t-l+u \rfloor} \sum_{m=1}^{\infty} q_m(l+n,l) \tag{S.274}$$

$$= \sum_{n=1}^{\lfloor t-l+u \rfloor} \Big(\mathcal{Q}_{(l+n,l)}(F(t,l+n)) + \sum_{k=1}^{\infty} q_k(l+n,l) \Big) \tag{S.275}$$

which means

$$F(t,l) = s(1-G(t-l,l)) \exp\Big[\sum_{n=1}^{\lfloor t-l \rfloor} \Big(\mathcal{Q}_{(l+n,l)}(F(t,l+n)) + \sum_{k=1}^{\infty} q_k(l+n,l) \Big) \Big] ... \tag{S.276}$$

$$+ \int_0^{t-l} \exp\Big[\sum_{n=1}^{\lfloor t-l+u \rfloor} \Big(\mathcal{Q}_{(l+n,l)}(F(t,l+n)) + \sum_{k=1}^{\infty} q_k(l+n,l) \Big) \Big] g(u,l)du \tag{S.277}$$

Finally, defining $\mathcal{Q}^*(s) := e^{\mathcal{Q}(s)}$ and turning the integral over g into a discrete sum, we have

$$F(t,l) = s(1-G(t-l,l)) \prod_{n=1}^{\lfloor t-l \rfloor} \mathcal{Q}^*(F(t,l+n)) e^{\sum_{k=1}^{\infty} q_k(l+n,l)} + \sum_{u=1}^{\lfloor t-l \rfloor} \prod_{n=1}^{\lfloor t-l+u \rfloor} \mathcal{Q}^*(F(t,l+n)) e^{\sum_{k=1}^{\infty} q_k(l+n,l)} g(u,l) \tag{S.278}$$

This matches very closely with the pgf in the discrete case, but has some extra terms as expected for the contradiction - firstly, the $\mathcal{Q}^*$ in place of the $\mathcal{Q}$, and also the extra $e^{\sum_{k=1}^{\infty} q_k}$ terms. When taking the small dt limit as in the previous subnote, these anomalies disappear, as

$$e^{\mathcal{Q}(s)} \sim e^{1+\alpha dt} \sim 1 + \alpha dt \sim \mathcal{Q}(s) \tag{S.279}$$

and

$$e^{\sum_{k=1}^{\infty} q_k(l+n,l)} \sim e^{\beta dt} \sim 1 \tag{S.280}$$

for some α and β. Thus, these dissimilarities only appear in the $O(dt^2)$ level (and hence disappear in the small dt limit). However, they will be non-trivial if dt is not small, underlining the importance of the assumption that N is continuous in probability - neglecting such an assumption could lead to materially wrong results in the case of a large step-size.

7.4 Discrete likelihood

If the epidemic happens in discrete time, it is significantly easier to calculate the likelihood. Define $A_{k,i}$ to be the number of infections caused at time k by a (still infectious) individual that was

infected at time i. Then, the number of infections which occur at time k is given by

$$\mathcal{A}_k(\boldsymbol{y}, \boldsymbol{d}) = \sum_{i=0}^{k-1} \sum_{j=1}^{y_i} A_{k,i}^j \mathcal{I}_{\{x_{ij} \leq k\}} \tag{S.281}$$

where each $A_{k,i}^j$ is an independent copy of $A_{k,i}$ and, similarly to before, x_{ij} is the time at which the jth individual infected at time i stops being infectious. Note that here, as previously in the discrete setting but in contrast to the continuous case, y_i can be zero.

Then, the likelihood is simply given by

$$L(\boldsymbol{y}, \boldsymbol{D}) = \left(\prod_{k=1}^{n} \mathbb{P}(\mathcal{A}_k(\boldsymbol{y}, \boldsymbol{d}) = y_k) \right) \left(\prod_{i=1}^{n} \prod_{j=1}^{y_i} g(x_{ij} - i, i) \right) \tag{S.282}$$

where, as we are in the discrete case, g is now a pmf. This gives a log-likelihood of

$$\ell(\boldsymbol{y}, \boldsymbol{D}) = \sum_{k=1}^{n} \log \left(\mathbb{P}(\mathcal{A}_k(\boldsymbol{y}, \boldsymbol{d}) = y_k) \right) + \sum_{i=1}^{n} \sum_{j=1}^{y_i} \log(g(x_{ij} - i, i)) \tag{S.283}$$

References

[1] D. Applebaum. *Lévy Processes and Stochastic Calculus.* en. Cambridge University Press, Apr. 2009.

[2] O. Barndorff-Nielsen and G. F. Yeo. “Negative binomial processes”. In: *J. Appl. Probab.* 6.3 (Dec. 1969), pp. 633–647.

[3] F. Bornemann. “Accuracy and stability of computing high-order derivatives of analytic functions by Cauchy integrals”. In: *Found. Comut. Math.* 11.1 (Feb. 2011), pp. 1–63.

[4] D. Champredon, J. Dushoff, and D. J. Earn. “Equivalence of the Erlang-distributed SEIR epidemic model and the renewal equation”. In: *SIAM Journal on Applied Mathematics* (2018). DOI: 10.1137/18M1186411.

[5] A. Cori et al. “A new framework and software to estimate time-varying reproduction numbers during epidemics”. In: *American Journal of Epidemiology* (2013). DOI: 10.1093/aje/kwt133.

[6] K. Crump and C. J. Mode. “A general age-dependent branching process. II”. en. In: *J. Math. Anal. Appl.* 25.1 (Jan. 1969), pp. 8–17.

[7] K. S. Crump and C. J. Mode. “A general age-dependent branching process. I”. In: *Journal of Mathematical Analysis and Applications* 24.3 (1968), pp. 494–508.

[8] W. Feller. “On the Integral Equation of Renewal Theory”. In: *The Annals of Mathematical Statistics* (1941). DOI: 10.1214/aoms/1177731708.

[9] C. Fraser. “Estimating individual and household reproduction numbers in an emerging epidemic”. In: *PLoS ONE* (2007). DOI: 10.1371/journal.pone.0000758.

[10] T. E. Harris et al. *The theory of branching processes*. Vol. 6. Springer Berlin, 1963.

[11] M. Kimmel. “The point-process approach to age- and time-dependent branching processes”. In: *Advances in Applied Probability* 15.1 (1983), pp. 1–20.

[12] J. N. Lyness. “Numerical algorithms based on the theory of complex variable”. In: *Proceedings of the 1967 22nd national conference*. ACM ’67. Association for Computing Machinery, Jan. 1967, pp. 125–133.

[13] J. C. Miller. “A primer on the use of probability generating functions in infectious disease modeling”. In: *Infectious Disease Modelling* 3 (2018), pp. 192–248.

[14] M. S. Pakkanen et al. “Unifying incidence and prevalence under a time-varying general branching process”. In: (July 2021). arXiv: 2107.05579 [q-bio.PE].
